# Danshensu for Myocardial Ischemic Injury: Preclinical Evidence and Novel Methodology of Quality Assessment Tool

**DOI:** 10.3389/fphar.2018.01445

**Published:** 2018-12-11

**Authors:** Xiao-yi Bao, Qun Zheng, Qiang Tong, Peng-chong Zhu, Zhuang Zhuang, Guo-qing Zheng, Yan Wang

**Affiliations:** ^1^Department of Cardiology, The Second Affiliated Hospital and Yuying Children's Hospital of Wenzhou Medical University, Wenzhou, China; ^2^Department of Neurology, The Second Affiliated Hospital and Yuying Children's Hospital of Wenzhou Medical University, Wenzhou, China

**Keywords:** danshensu, infarct size, ischemia, reperfusion, meta-analysis, methodology

## Abstract

**Background:** Danshensu (DSS) possesses unique bioactivity on the cardiovascular system. However, there is a lack of systematical summary of DSS for acute myocardial ischemia injury and no quality assessment tool for the systematical review of cell experiments. Here, we aimed to assess the preclinical evidences and possible mechanisms of DSS for myocardial ischemia injury, and to develop a quality assessment tool for the systematical review of cell experiments.

**Methods:** Thirty-two studies with 473 animals and 134 cells were identified by searching seven databases. All data analysis was performed using RevMan 5.3. CAMARADES 10-item checklist was used to assess the methodological quality of animal experiments. A new 10-item checklist was first developed to assess the methodological quality of cell studies.

**Results:** The score of study quality ranged from 3 to 7 points in animal studies, while the cell studies scored 3–6 points. Meta-analysis showed that DSS had significant effects on reducing myocardial infarct (MI) size *in vivo*, and increasing cell viability and reducing apoptosis rate *in vitro* compared with controls (*P* < 0.01). The possible mechanisms of DSS for MI are improving circulation, antioxidant, anti-apoptosis, anti-inflammatory, promoting angiogenesis, anti-excessive autophagy, anti-calcium overload, and improving energy metabolism.

**Conclusions:** DSS could exert cardioprotective effect on myocardial ischemia injury, and thus is a probable candidate for further clinical trials andtreatment of AMI. In addition, the newly devloped 10-item checklist for assessing methodological quality of cell study that recommened to use the sysmatic review of cell studies.

## Introduction

Acute myocardial infarction (AMI) is a major cause of death and disability worldwide (Mozaffarian et al., 2016). During AMI, the damage inflicted on the myocardium results in two processes: ischemia and the following reperfusion (I/R) (Ibanez et al., 2015). Thus, timely myocardial reperfusion using either thrombolytic therapy or primary percutaneous coronary intervention (PPCI) is the most effective therapy for limiting myocardial infarct (MI) size, preserving left-ventricular systolic function and reducing the onset of heart failure (Frohlich et al., 2013). However, the mortality and morbidity of patients remain significant with 9% death and 10% heart failure at 1 year after AMI. The fact that a therapeutic intervention administered solely at the time of myocardial I/R injury can reduce MI size by up to half only suggests that myocardial I/R injury may account for up to 50% of the final MI size (Bulluck et al., 2016). Although the process of myocardial reperfusion has been optimized by advances in stent technology, new antiplatelet drugs and novel antithrombotic agents, the studies on drug for necrosis and inflammation of cardiac myocytes during AMI made little evident progress. And there is still no effective therapy for preventing myocardial I/R injury in PPCI patients (Hausenloy and Yellon, 2013). Thus, it is urgent to seek new cardioprotective strategies to improve myocardial salvage and cardiac function when AMI happens.

Phenolic and flavonoid compounds with specific structures exhibit strong activity. For example, agrimonolide, curcumin, emodin, resveratrol, baicalin and meletin exerted anti-inflammatory, antioxidant, anti-apoptosis effects and regulated metabolism through multiple intracellular signaling pathways (Chen et al., 2016, 2018a; Teng et al., 2016a,b). Previous studies showed that the application of these compounds is beneficial to various diseases (Chen et al., 2018b; Teng and Chen, 2018). Danshen root, Radix Salviae Miltiorrhizae, the dried root of Salvia miltiorrhiza, has been widely used to treat various vascluar diseases including coronary heart disease worldwide (Zhang et al., 2018). Previous studies showed that one of the main water-soluble phenolic components from Danshen, Danshensu (DSS) [(3-(3,4-dihydroxyphenyl)-lactic acid)], exerted potential cardiovascular protective effects both *in vitro* and *in vivo* (Dong et al., 2009; Cui et al., 2014; Yu J. et al., 2015; Li et al., 2016; Yin et al., 2017; Zhang X. et al., 2017). The specific chemical structures of Danshensu and its derivatives are listed in Table 1. Futhermore, preclinical systematic review can evaluate the efficacy and mechanism of drugs more systematically, establish a test field for further animal experiments, provide reliable information for drug research, and lay a foundation for future clinical research (Sena et al., 2014). what's more, there are no quality assessment tools for a systematical review of cell experiments. Thus, we aimed to provide the preclinical evidence by assessing the efficacy and mechanisms of DSS for expermental AMI, and develop a quality assessment tool for a systematical review of cell experiments.

**Table 1:** Specific chemical structures of Danshensu and its derivatives.

Danshensu	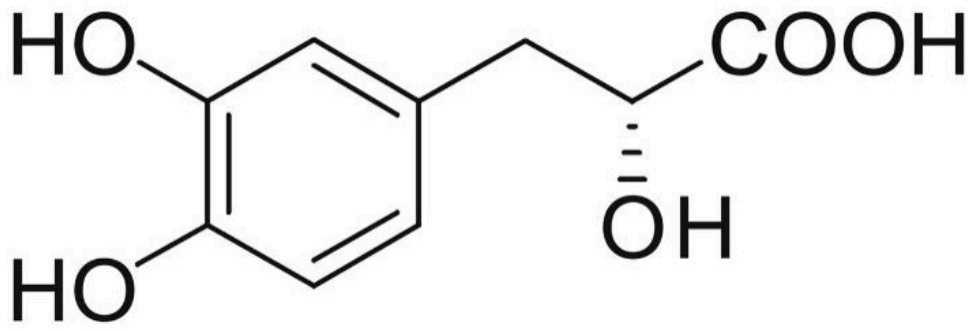	Yin et al., 2017
		Li et al., 2016
		Yu J. et al., 2015
		Jiang et al., 1982
		Tang et al., 1989
		Li et al., 1996
		Zhang et al., 2010
		Lu et al., 2010
		Li et al., 2012
		Yin et al., 2013
		Wei et al., 2016
		Song et al., 2016
		Fan et al., 2016
		Hu et al., 2016
		Gao et al., 2017
		Zhu et al., 1999
		Guo et al., 2006
		Meng et al., 2016
		Quan et al., 2012
Danshensu derivative	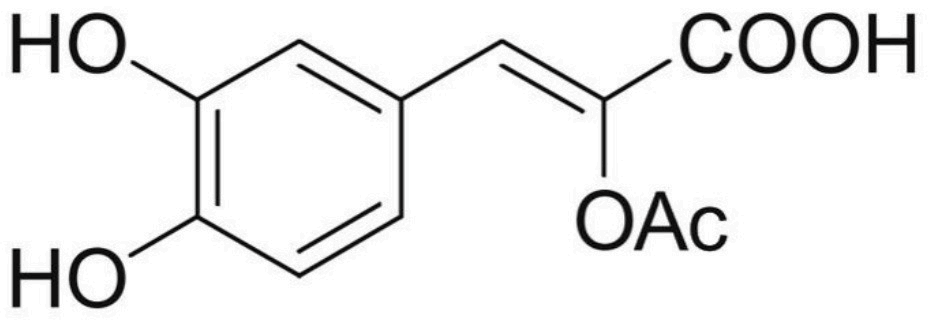	Dong et al., 2009
	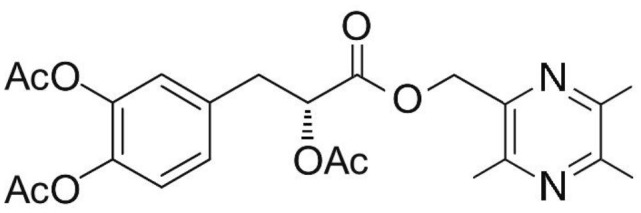	Cui et al., 2014
		Zhao et al., 2012
		Cui G. et al., 2013
	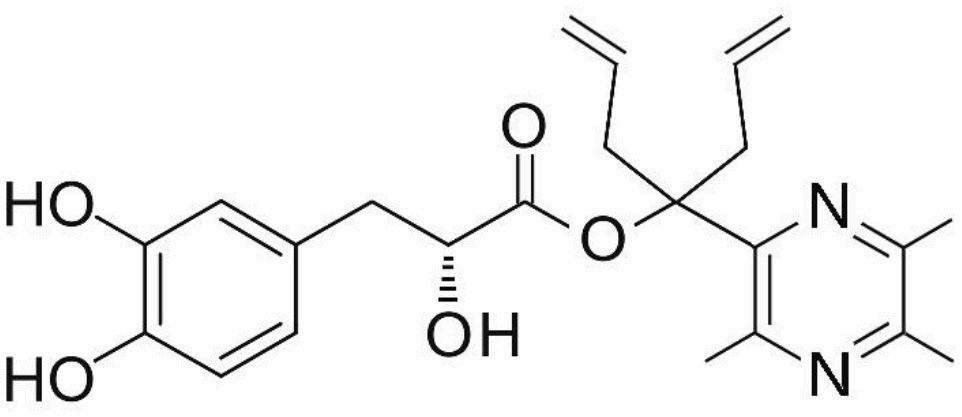	Zhang X. et al., 2017
	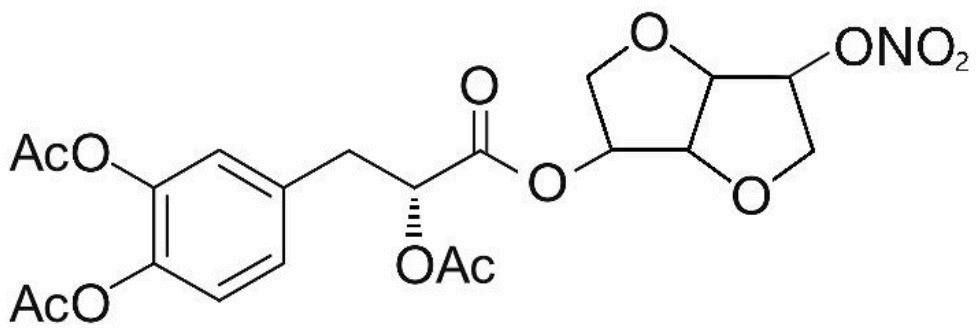	Xiang et al., 2009
	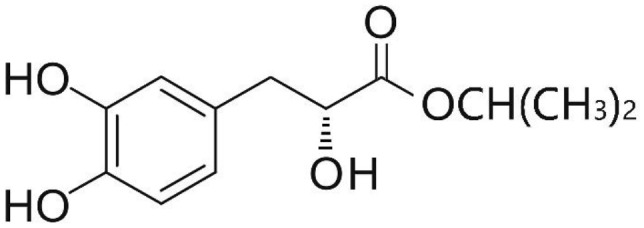	Cheng et al., 2010
	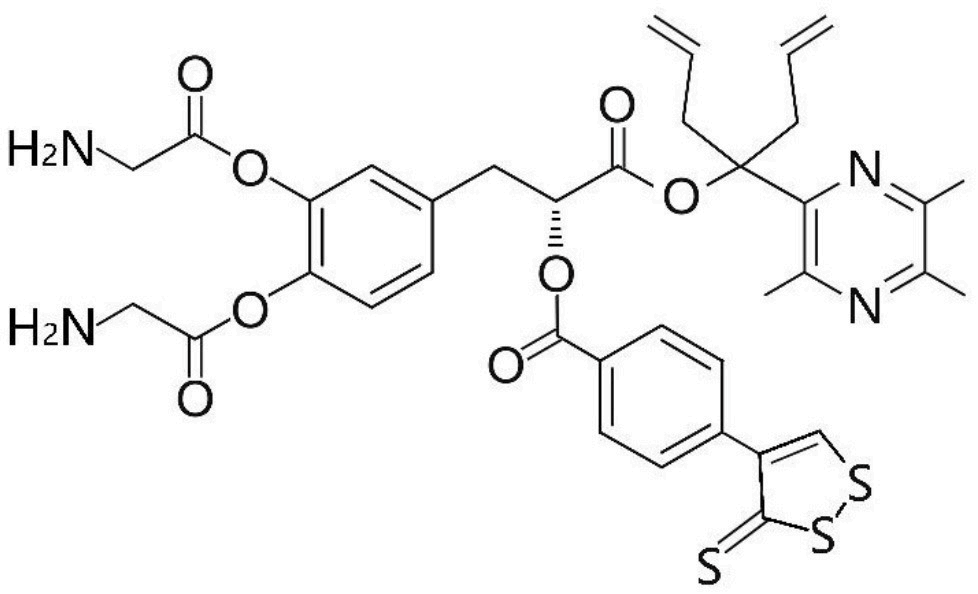	Wang et al., 2016
	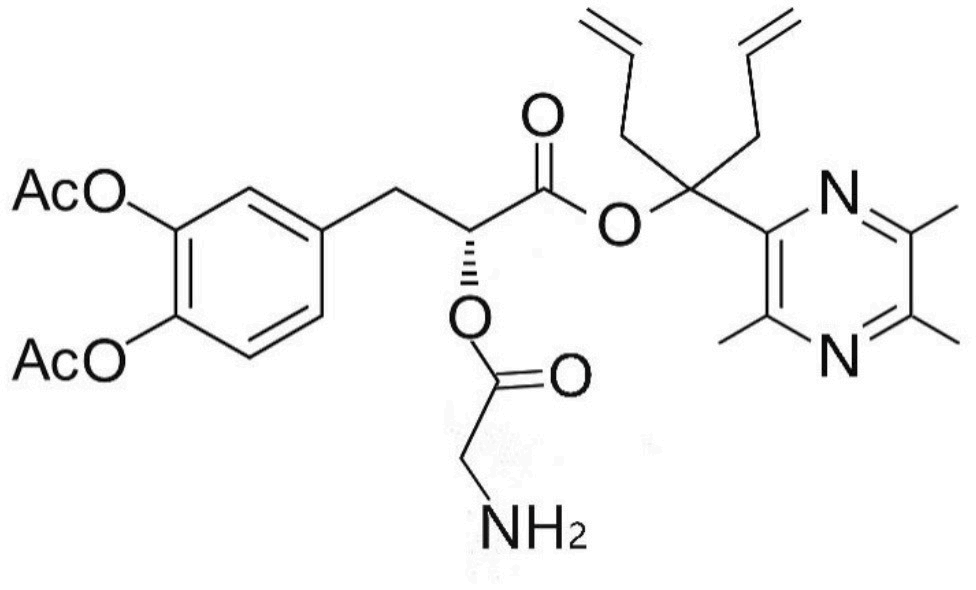	Wang et al., 2017
	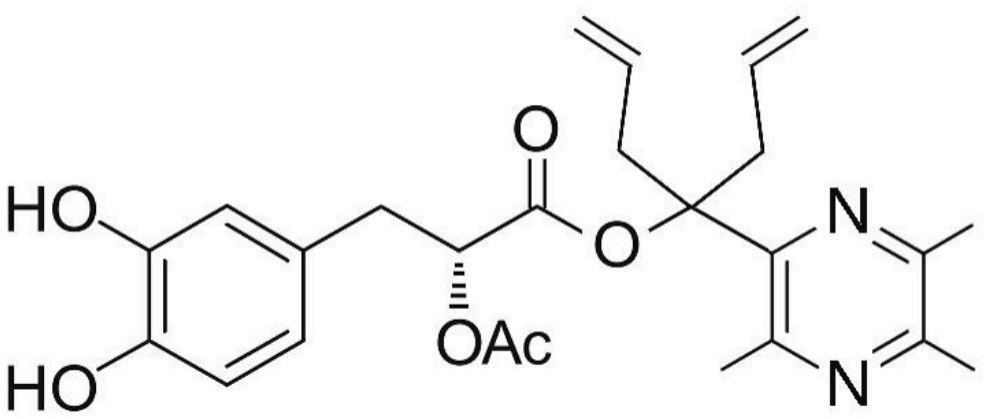	Tang et al., 2018
	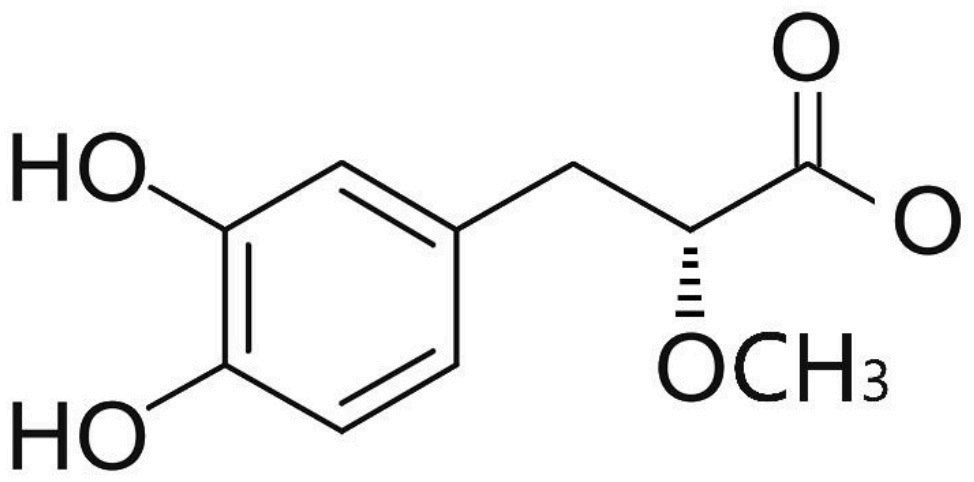	Cui Q. B. et al., 2013
	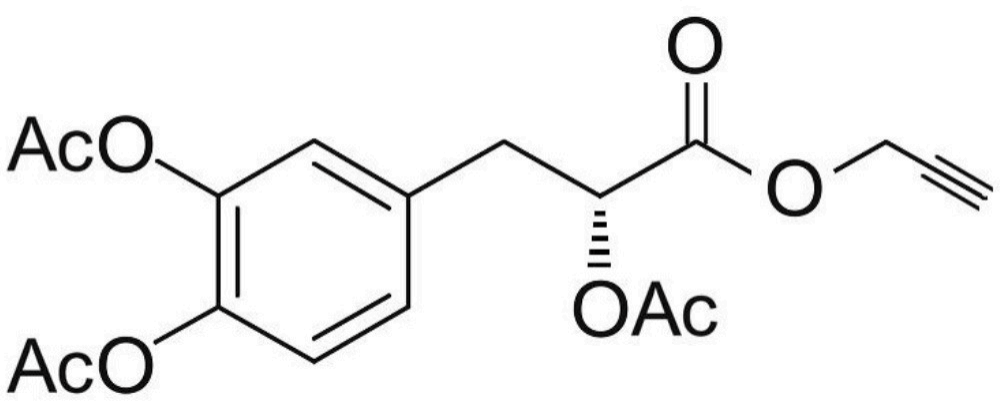	Xu et al., 2013
	Flexible nano-liposom of DSS (The specific chemical structure is unknown)	Chen et al., 2014

## Methods

The prensent study was conducted according to the Preferred Reporting Items for Systematic reviews and Meta-Analyses (PRISMA) guidelines (Liberati et al., 2009).

### Search Strategies

The following databases were searched from the inception to February, 2018. They are PubMed, CENTRAL, Web of Science, Chinese National Knowledge Infrastructure, Wanfang Data Information Site, VIP information database and Chinese Biomedical Literature Database. The keywords used are as follows: (1) “Danshensu OR Salvianic acid A OR tanshinol OR 3,4-dihydroxyphenyl” AND “myocardial infarction OR myocardial ischemia OR myocardial I/R” limited on animals; (2) “Danshensu OR Salvianic acid A OR tanshinol OR 3,4-dihydroxyphenyl” AND “hypoxia OR injury OR damage OR impair” limited on cells. The literature search in this article was last updated in February, 2018.

### Eligibility Criteria

Inclusion criteria were prespecified as follows: (1) experimental animal models of myocardial ischemia or myocardial cell injury models; (2) analyzed treatments received the DSS [to enhance stability and activities of danshensu, Danshensu derivatives (Dong et al., 2009; Cui et al., 2014; Zhang X. et al., 2017) have been widely developed and thus was also included]; (3) comparator interventions received vehicle or non-functional liquid with equal volume or conventional medicine or no treatment; (4) the primary outcome measures were MI size in animal model, and the viability and/or apoptosis rate in cell model. The second outcome measures were biochemical markers of MI, left ventricular ejection fraction (LVEF), left ventricular shortening fraction (LVFS), and/or level of ST-segment depression, and mechanism indices. Exclusion criteria were prespecified as follows: (1) experimental models unrelated to myocardial ischemic injury; (2) the use of DSS in combination with other drugs in treatment group; (3) no control group or comparing DSS with other herbs in control group; (4) duplicate publication; (5) case report, clinical trial and review article.

### Data Extraction

The detailed information of included studies were collected by two indenpendent authors. For animal studies, standard extraxted form on the basis of our previous publication was adopted (Zhang K. J. et al., 2017). For cell studies, the information extracted from included studies was as follows: (1) the first author's name and year of publication; (2) the features of cells, such as name, source (organism, age, tissue) and primary/subcultured; (3) model of cell injury; (4) the information of treatment/control group, including therapeutic drug, dosage, method of administration, duration of treatment; (5) mean value and standard deviation of outcomes.

If the outcomes were displayed through multiple time points, only the peak time point was included. Only the data of highest dose was selected, if the experimental group included gradient doses of drug therapy. Owing to some data were only displayed by graphics, the correspondence authors were be contacted for further information. If there were no responding, the data in the graph were measured by the digital ruler software.

### Risk of Bias in Individual Studies

The risk of bias (ROB) in animal studies was refered to the ten-item scale using our previously publications (Zhang K. J. et al., 2017). Owing to absence of the ROB assessment scale in cell experiments, we developed a novel 10-item checklist as follows: (A) peer reviewed publication; (B) use appropriate cells to study; (C) cell lines with reliable source or validated by appropriate methods; (D) assess toxicity of treatment on cells; (E) culture environment (culture media/sera, pH/CO_2_ and temperature); (F) random allocation to treatment or control; (G) blinded induction of model; (H) blinded assessment of outcome; (I) calculation of the sample size necessary to achieve sufficient power; and (J) statement of potential conflict of interests. Each item was awarded one point. All studies were evaluated by two independent authors. Possible divergences in the evaluation process were properly addressed through contacting with correspondence authors.

### Statistical Analysis

All data analysis was implemented by RevMan 5.3. We calculated the standard mean difference (SMD) with 95% Confidence Intervals (CIs). Heterogeneity was assessed using the Cochrane Q-statistic test and the *I*^2^-statistic test. When *I*^2^ > 50%, indicating substantial heterogeneity and a random-effects model was adopted. Conversely, a fixed-effects model was used. Sources of heterogeneity were searched as far as possible and subgroup analysis was carried out when necessary. Funnel plots were used to intuitively reflect publication bias when more than ten studies were identified. Sensitivity analysis was carried out to prove the robustness of our main results. *P* < 0.05 was considered statistically significant.

## Results

### Study Selection

**Figure 1:**
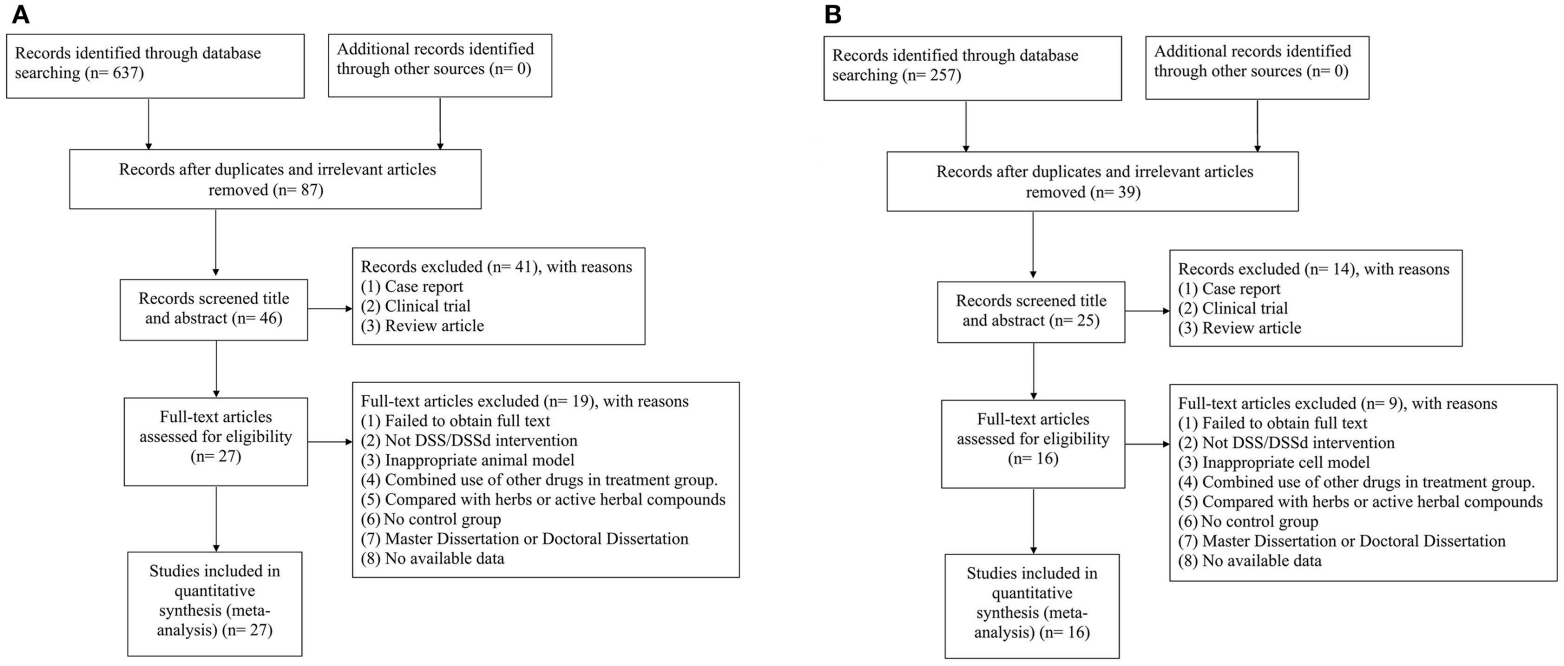
**(A)** Search strategy for animal experiments: 637 potentially relevant studies were identified, after removal of duplicates and the application of inclusion and exclusion criteria, 27 studies were included in the meta-analysis; **(B)** search strategy for cell experiments: 257 potentially relevant studies were identified, after removal of duplicates and the application of inclusion and exclusion criteria, 16 studies were included in the meta-analysis.

For animal studies, 637 potentially relevant studies were identified through database searching, of which 550 were duplicated. After preliminary screening of the titles and abstracts, we excluded 41 studies because of case report, clinical observation, and review article. Then secondary screening was conducted by reading the full text of the remaining 46 studies, and 19 studies were excluded because of at least one of the following reasons: (1) failed to obtain full text; (2) not DSS intervention; (3) inappropriate animal model; (4) combined use of other drugs in treatment group; (5) compared with herbs or active herbal compounds; (6) no control group; (7) master dissertation or doctoral dissertation; (8) no available data. Ultimately, 27 eligible studies (Jiang et al., 1982; Tang et al., 1989, 2018; Li et al., 1996, 2012, 2016; Dong et al., 2009; Xiang et al., 2009; Cheng et al., 2010; Lu et al., 2010; Zhang et al., 2010; Quan et al., 2012; Zhao et al., 2012; Cui G. et al., 2013; Yin et al., 2013, 2017; Chen et al., 2014; Cui et al., 2014; Yu J. et al., 2015; Fan et al., 2016; Hu et al., 2016; Song et al., 2016; Wang et al., 2016, 2017; Wei et al., 2016; Gao et al., 2017; Zhang X. et al., 2017) were selected (Figure 1A).

For cell studies, 257 potentially relevant studies were identified through database searching, of which 39 were duplicated. After preliminary screening of the titles and abstracts, we excluded 14 studies because of case report, clinical observation, and review article. Then secondary screening was conducted by reading the full text of the remaining 25 studies, nine studies were excluded because of: (1) failed to obtain full text; (2) not DSS intervention; (3) inappropriate cell model; (4) combined use of other drugs in treatment group; (5) compared with herbs or active herbal compounds; (6) no control group; (7) master dissertation or doctoral dissertation; (8) no available data. Ultimately, 16 eligible studies (Zhu et al., 1999; Guo et al., 2006; Dong et al., 2009; Zhao et al., 2012; Cui G. et al., 2013; Cui Q. B. et al., 2013; Xu et al., 2013; Yin et al., 2013; Fan et al., 2016; Hu et al., 2016; Meng et al., 2016; Wang et al., 2016, 2017; Gao et al., 2017; Zhang X. et al., 2017; Tang et al., 2018) were selected (Figure 1B).

### Characteristics of Included Studies

#### Animal Experiments

Twenty-seven studies were involved in animal experiments. Eleven studies were published in chinese (Jiang et al., 1982; Tang et al., 1989; Li et al., 1996; Xiang et al., 2009; Cheng et al., 2010; Lu et al., 2010; Zhang et al., 2010; Quan et al., 2012; Zhao et al., 2012; Chen et al., 2014; Wei et al., 2016), and the rest was published in English (Dong et al., 2009; Li et al., 2012, 2016; Cui G. et al., 2013; Yin et al., 2013, 2017; Yu J. et al., 2015; Fan et al., 2016; Hu et al., 2016; Song et al., 2016; Wang et al., 2016, 2017; Gao et al., 2017; Zhang X. et al., 2017; Tang et al., 2018) between 1982 and 2018. Sprague-Dawley (SD) male rats were used in 21 studies (Dong et al., 2009; Xiang et al., 2009; Lu et al., 2010; Zhang et al., 2010; Li et al., 2012, 2016; Quan et al., 2012; Zhao et al., 2012; Cui G. et al., 2013; Yin et al., 2013, 2017; Chen et al., 2014; Cui et al., 2014; Yu J. et al., 2015; Fan et al., 2016; Hu et al., 2016; Song et al., 2016; Wei et al., 2016; Gao et al., 2017; Wang et al., 2017; Zhang X. et al., 2017), two that used male Wistar rats (Tang et al., 1989; Li et al., 1996), one that used both female and male Wistar rats (Cheng et al., 2010), two that used Zebrafish embryos (Wang et al., 2016; Tang et al., 2018) and one that used male canines with unknow species (Jiang et al., 1982). The weight of the rats were 180–320 g, and the canines were 12–26 kg. To induce anesthesia, 16 studies used nembutal (Jiang et al., 1982; Tang et al., 1989; Li et al., 1996, 2012, 2016; Xiang et al., 2009; Cheng et al., 2010; Quan et al., 2012; Yin et al., 2013, 2017; Chen et al., 2014; Yu J. et al., 2015; Fan et al., 2016; Hu et al., 2016; Wang et al., 2017; Zhang X. et al., 2017) seven studies used chloral hydrate (Dong et al., 2009; Zhang et al., 2010; Zhao et al., 2012; Cui G. et al., 2013; Cui et al., 2014; Wei et al., 2016; Gao et al., 2017); and two studies used ethyl carbamate (Lu et al., 2010; Song et al., 2016). Fifteen MI models were produced by ligation of the left anterior descending coronary artery (LAD) (Jiang et al., 1982; Dong et al., 2009; Xiang et al., 2009; Lu et al., 2010; Quan et al., 2012; Zhao et al., 2012; Cui G. et al., 2013; Yin et al., 2013, 2017; Chen et al., 2014; Cui et al., 2014; Hu et al., 2016; Wei et al., 2016; Wang et al., 2017; Zhang X. et al., 2017) seven models produced by the langendorff method (Tang et al., 1989; Li et al., 1996; Cheng et al., 2010; Zhang et al., 2010; Yu J. et al., 2015; Fan et al., 2016; Gao et al., 2017); three models produced by subcutaneous injection of Isoproterenol (ISO) (Li et al., 2012, 2016; Song et al., 2016); and the remain two by exposure to Doxorubicin (Dox) (Wang et al., 2016; Tang et al., 2018). For outcome measures, 16 studies reported the MI size (Jiang et al., 1982; Dong et al., 2009; Xiang et al., 2009; Lu et al., 2010; Li et al., 2012; Quan et al., 2012; Cui G. et al., 2013; Yin et al., 2013, 2017; Chen et al., 2014; Cui et al., 2014; Yu J. et al., 2015; Hu et al., 2016; Wei et al., 2016; Wang et al., 2017; Zhang X. et al., 2017) level of ST-segment change (Li et al., 2012), LVEF or LVFS (Yin et al., 2017) was reported in one study, respectively. Lactate dehydrogenase (LDH) was reported in eight studies (Dong et al., 2009; Xiang et al., 2009; Lu et al., 2010; Li et al., 2012; Cui et al., 2014; Yu J. et al., 2015; Fan et al., 2016; Song et al., 2016), creatine kinase (CK) in seven studies (Dong et al., 2009; Xiang et al., 2009; Lu et al., 2010; Cui et al., 2014; Yu J. et al., 2015; Fan et al., 2016; Song et al., 2016), creatine kinase-MB (CK-MB) in five studies (Li et al., 2012, 2016; Quan et al., 2012; Yin et al., 2013; Hu et al., 2016), cardiac troponin-T (cTnT) in one study (Li et al., 2016). The overall characteristics of included publications are shown in Table 2.

**Table 2:** Characteristics of the 27 included animal studies.

**Study (years)**	**Species (Sex,** ***n* = experimental/** **control group)**	**Weight**	**Model (method)**	**Anesthetic**	**Treatment group (Method to astragal sides)**	**Control group (Negative/positive)**	**Outcome index (time)**	**Intergroup differences**
Jiang et al., 1982	? canines (male, 11/14)	12–26 kg	Block LAD	Nembutal	DSS (8 mg/kg, *i.v*) After ischemia	No treatment	1. Myocardial infarct size	1. *P* < 0.01
Tang et al., 1989	Wistar rats (male, 6/6)	200–250 g	Langendorff Model 10 min stabilization I/R (60 min/20 min)	Nembutal (0.6%)	DSS (4*10^−8^ g/ml) during stabilization	No treatment	1. SOD 2. GSH-Px	1. *P* < 0.001 2. *P* < 0.005
Li et al., 1996	Wistar rats (male, 7/7)	250–280 g	Langendorff Model 10 min stabilization I/R (40 min/20 min)	Nembutal (0.6%)	DSS (0.4 mg/mL) during stabilization and reperfusion	No treatment	1. ATP 2. TAN 3. EC	1. *P* < 0.001 2. *P* < 0.001 3. *P* < 0.004
Zhang et al., 2010	SD rats (male, 6/6)	280–320 g	Langendorff Model 30 min stabilization I/R (30 min/30 min)	Chloral hydrate (10%)	DSS (200 um) at the beginning of ischemia	No treatment	1. ATP 2. EC	1. *P* < 0.01 2. *P* < 0.01
Lu et al., 2010	SD rats (male, 10/10)	200–220 g	Block LAD	Urethane (20%)	DSS (24 mg/kg/day, *i.p*) for 7 days before ischemia	0.9% normal saline (10 ml/kg/day, *i.p*) for 7 days before ischemia	1. Myocardial infarct size 2. CK 3. LDH	1. *P* < 0.01 2. *P* < 0.01 3. *P* < 0.01
Li et al., 2012	SD rats (male, 8/8)	200–240 g	ISO (85mg/kg/day, i.h) for 2 consecutive days	Nembutal (35 mg/kg)	DSS (160 mg/kg/day, *i.g*) for 21 days and ISO on 20th and 21st day	Isosteric 0.3% CMC-Na solution (*i.g*) for 21 days and ISO on 20th and 21st day	1. Myocardial infarct size 2. ΔST 3. Heart weight/body weight ratio 4. CK-MB 5. LDH 6. SOD 7. TBARS 8. GSH 9. GST 10. Nrf2 11. Bax 12. Bcl-2 13. Caspase3	1. *P* < 0.01 2. *P* < 0.001 3. *p* < 0.001 4. *P* < 0.001 5. *p* < 0.001 6. *p* < 0.001 7. *P* < 0.001 8. *p* < 0.001 9. *p* < 0.001 10. *P* < 0.05 11. *p* < 0.001 12. *p* < 0.001 13. *p* < 0.001
Yin et al., 2013	SD rats (male, 8/8)	230–270 g	Block LAD for 30 min then reflow for 180 min	Nembutal (40mg/kg)	DSS (60 mg/kg, *i.v*) Within 5 min at the beginning of reperfusion	Isasteric normal saline (*i.v*) Within 5 min at the beginning of reperfusion	1. Myocardial infarct size 2. CK-MB	1. *P* < 0.05 2. *P* < 0.01
Yu J. et al., 2015	SD rats (male, 8/8)	280–320 g	Langendorff Model 30 min stabilization I/R (30 min/30 min)	Nembutal (30 mg/kg)	DSS (10 um) 10 min before ischemia	No treatment	1. Myocardial infarct size 2. CK 3. LDH 4. ROS 5. MDA 6. SOD 7. GSH-Px 8. SOD mRNA 9. HO-1 mRNA 10. Nrf2 mRNA 11. P-AKT 12. P-ERK 13. Nrf2	1. *P* < 0.01 2. *P* < 0.01 3. *P* < 0.05 4. *P* < 0.01 5. *P* < 0.01 6. *P* < 0.01 7. *P* < 0.05 8. *P* < 0.01 9. *P* < 0.01 10. *P* < 0.01 11. *P* < 0.01 12. *P* < 0.01 13. *P* < 0.01
Wei et al., 2016	SD rats (male, 12/12)	220–260 g	Block LAD	Chloral hydrate (10%, 0.4 ml/kg)	DSS (2 mg/kg/day, *i.v*) for 7 days after ischemia	Isasteric normal saline (*i.v*) for 7 days afterischemia	1. Myocardial infarct size 2. MVD 3. VEGF 4. SDF-1 5. CXCR4	1. *P* < 0.01 2. *P* < 0.01 3. *P* < 0.05 4. *P* < 0.01 5. *P* < 0.01
Song et al., 2016	SD rats (male, 8/8)	180–220 g	ISO (85 mg/kg/day, i.h) for 2 consecutive days	Urethane (4 mg/kg)	DSS (10 mg/kg/day, *i.p*) for 7 days before ischemia	Equivalent distilled water for 7 days before ischemia	1. CK 2. LDH	1. *P* < 0.01 2. *P* < 0.01
Fan et al., 2016	SD rats (male, 8/8)	280–320 g	Langendorff Model 30 min stabilization I/R (30 min/60 min)	Nembutal (50 mg/kg)	DSS (10 um) during reperfusion	No treatment	1. CK 2. LDH 3. Bcl-2 4. Bax 5. Caspase-3 6. P-mTOR mRNA 7. P-62 mRNA 8. Beclin-1 mRNA 9. LC3 mRNA 10. P-mTOR 11. P-s6k1 12. P-s6 13. P-62 14. Beclin-1 15. LC3-II 16. Bcl-2 mRNA 17.BaxmRNA 18.Caspase-3mRNA	1. *P* < 0.05 2. *P* < 0.05 3. *P* < 0.01 4. *P* < 0.01 5. *P* < 0.01 6. *P* < 0.05 7. *P* < 0.01 8. *P* < 0.01 9. *P* < 0.01 10. *P* < 0.05 11. *P* > 0.05 12. *P* > 0.05 13. *P* < 0.01 14. *P* < 0.01 15. *P* > 0.05 16. *P* < 0.01 17. *P* < 0.01 18. *P* < 0.01
Li et al., 2016	SD rats (male, 8/8)	200–240 g	ISO (85 mg/kg/day, i.h) for 2 consecutive days	Nembutal (35mg/kg)	DSS (160 mg/kg/day, *i.g*) for 21 days and ISO on 20th and 21st day	Isosteric 0.3% CMC-Na solution (*i.g*) for 21 days and ISO on 20th and 21st day	1. CK-MB 2. cTnT 3. ROS 4. TBARS 5. GSH/GSSH 6. Nrf2 7. Keap1 8. HO-1 9. GST 10. Cytochrome C 11. TNF-α 12. Bcl-2 13. Bax 14. Caspase-3 15. P-PI3K/PI3K 16. P-Akt/Akt	1. *P* < 0.001 2. *P* < 0.001 3. *P* < 0.001 4. *p* < 0.001 5. *p* < 0.001 6. *P* < 0.001 7. *P* < 0.001 8. *P* < 0.001 9. *P* < 0.001 10. *P* < 0.001 11. *P* < 0.01 12. *P* < 0.01 13. *P* < 0.001 14. *p* < 0.001 15. *P* < 0.001 16. *P* < 0.001
Hu et al., 2016	SD rats (male,9/9)	230–270 g	Block LAD for 30 min then reflow for 180 min	Nembutal (40 mg/kg)	DSS (60 mg/kg, *i.v*) at the time of reperfusion	No treatment	1. Myocardial infarct size 2. CK-MB 3. MDA 4. SOD	1. *P* < 0.05 2. *P* < 0.01 3. *P* < 0.05 4. *P* < 0.05
Yin et al., 2017	SD rats (male, 17/17)	230–270 g	Block LAD	Nembutal (30mg/kg)	DSS (60 mg/kg/day, *i.v*) for 14 days after 24 h of ischemia	Isasteric normal saline (*i.v*) for 14 days after 24 h of ischemia	1. Myocardial infarct size 2. MVD 3. VEGF 4. bFGF 5. SDF-1 6. CXCR-4 7. LVEF 8. LVFS	1. *P* < 0.01 2. *P* < 0.01 3. *P* < 0.05 4. *P* < 0.05 5. *P* < 0.05 6. *P* < 0.01 7. *P* < 0.05 8. *P* < 0.05
Gao et al., 2017	SD rats (male, 15/15)	220–280 g	Langendorff Model 30 min stabilization I/R (30 min/60 min)	Chloral hydrate (300 mg/kg)	DSS (10 um) during reperfusion	No treatment	1. Cardiac apoptosis 2. Caspase-3 3. MMP 4. MPTP 5. ATP 5G1 mRNA 6. ATP 5G1	1. *P* < 0.01 2. *P* < 0.05 3. *P* < 0.05 4. *P* < 0.05 5. *P* < 0.01 6. *P* < 0.01
Dong et al., 2009	SD rats (male, 6/6)	200–250 g	Block LAD	Chloral hydrate (10%, 60mg/kg)	DSS (60 mg/kg/day, *i.p*) for 7 days beforeischemia and 2 days afterischemia	No treatment	1. Myocardial infarct size 2. CK 3. LDH 4. MDA 5. SOD	1. *P* < 0.05 2. *P* < 0.05 3. *P* < 0.05 4. *P* < 0.05 5. *P* < 0.05
Xiang et al., 2009	SD rats (male, 7/8)	200–250 g	Block LAD for 30 min then reflow for 180 min	Nembutal (40 mg/kg)	DSS (9.47 mg/kg, ?) 2 h before ischemia	No treatment	1. Myocardial infarct size 2. CK 3. LDH 4. NO 5. MDA	1. *P* < 0.01 2. *P* < 0.01 3. *P* < 0.05 4. *P* < 0.01 5. *P* < 0.01
Cheng et al., 2010	Wistar rats (male, female, 7/7)	290–320 g	Langendorff Model 10 min stabilization I/R (40 min/20 min)	Nembutal (1%)	DSS (0.4 mg/L) during reperfusion	No treatment	1. ATP 2. TAN	1. *P* < 0.05 2. *P* < 0.01
Zhao et al., 2012	SD rats (male, 10/10)	180–220 g	Block LAD	Chloral hydrate (10%, 3.5 ml/kg)	DSS (20 mg/kg, *i.v*) 15 min after ischemia	Isasteric normal saline (*i.v*) 15 min after ischemia	1. Bcl2/Bax 2. Caspase-3	1. *P* < 0.01 2. *P* < 0.01
Quan et al., 2012	SD rats (male, 7/7)	230–270 g	Block LAD for 30 min then reflow for 180 min	Nembutal (3%)	DSS (60 mg/kg, *i.v*) at the time of reperfusion	Isasteric normal saline (*i.v*) at the time of reperfusion	1. Myocardial infarct size 2. CK-MB 3. IL-1 4. IL-6 5. TNF-α	1. *P* < 0.05 2. *P* < 0.05 3. *P* < 0.05 4. *P* < 0.05 5. *P* < 0.05
Cui G. et al., 2013	SD rats (male, 8/8)	220–250 g	Block LAD for 30 min then reflow for 120 min	Chloral hydrate (10%)	DSS (20 mg/kg, *i.v*) 15 min after ischemia	Isasteric normal saline (*i.v*) 15 min after ischemia	1. Myocardial infarct size 2. P-PI3K/PI3K 3. P-Akt/Akt 4. Nrf2 5. HO-1	1. *P* < 0.05 2. *P* < 0.05 3. *P* < 0.05 4. *P* < 0.05 5. *P* < 0.05
Chen et al., 2014	SD rats (male, 10/10)	220–300 g	Block LAD for 10 min, reflow for 24 min, then block for 20 min, reflow for 80 min	Nembutal (45 mg/kg)	DSS patch (0.1 g/kg) for 3 days before r ischemia	Normal patch for 3 days before modeling	1. Myocardial infarct size	1. *P* < 0.05
Cui et al., 2014	SD rats (male, 15/18)	220–250 g	Block LAD	Chloral hydrate (10%)	DSS (30 mg/kg, *i.p*) 15 min after ischemia	Isasteric normal saline (*i.p*) 15 min after ischemia	1. Myocardial infarct size 2. CK 3. LDH 4. MDA 5. SOD	1. *P* < 0.01 2. *P* < 0.05 3. *P* < 0.05 4. *P* < 0.01 5. *P* < 0.01
Wang et al., 2016	Zebrafish embryos (–, 3/3)	–	Treated with Dox (1 μM)	–	DSS (30 μM) for 32 h	No treatment	1. Stroke volume 2. Cardiac output 3. Fractional shortening	1. *P* < 0.01 2. *P* < 0.01 3. *P* < 0.01
Zhang X. et al., 2017	SD rats (male, 9/9)	250–270 g	Block LAD for 30 min then reflow for 180 min	Nembutal (5%)	DSS (90 mg/kg, ?) 30 min before ischemia and at the time of reperfusion	No treatment	1. Myocardial infarct size	1. *P* < 0.001
Wang et al., 2017	SD rats (male, 7/7)	250–270 g	Block LAD	Nembutal (5%)	DSS (30 mg/kg, *i.v*) 10 min after ischemia	Isasteric normal saline (*i.v*) 10 min after ischemia	1. Myocardial infarct size	1. *P* < 0.01
Tang et al., 2018	Zebrafish embryos (–, 3/3)	–	Treated with Dox (35 μM)	–	DSS (30 μM) for 24 h	No treatment	1. Stroke volume 2. Cardiac output 3. Fractional shortening	1. *P* < 0.01 2. *P* < 0.01 3. *P* < 0.01

*SD rats, Sprague-Dawley; LAD, the left anterior descending coronary artery; DSS, Danshensu; LVEF, left ventricular ejection fraction; LVFS, left ventricular shortening fraction; ΔST, level of ST-segment depression; CK, creatine kinase; LDH, lactate dehydrogenase; CK-MB, creatine kinase-MB; cTnT, cardiac troponin T; P-Akt, phosphothreonine kinase; P-PI3K, phosphatidylinositol 3-kinase; P-ERK, phosphorylated extracellular signal-regulated kinases; ROS, reactive oxygen species; Nrf2, nuclear factor erythroid 2-related factor 2; HO-1, heme oxygenase-1; KEAP1, Kelch-like ECH-associated protein 1; GST, glutathione-S-transferase; GSH-Px, glutathione peroxidase; GSH, glutathione synthetase; MDA, malondialdehyde; SOD, superoxide dismutase; TBARS, thiobarbituric acid reactive substances; Bcl-2, B-cell lymphoma-2; Bax, BCL2-associated X protein; TNF-α, tumor necrosis factor-α; IL-1, Interleukin-1; IL-6, Interleukin-6; SDF-1α, stromal cell-derived factor-1 α; CXCR4, C-X-C chemokine receptor type 4; VEGF, vascular endothelial growth factor; MVD, microvesseldensity; bFGF, basic fibroblast growth factor; NO, nitric oxide; P-mTOR, phosphorylated mammalian target of rapamycin; P-S6k1, phosphorylated ribosomal protein S6 kinase beta-1; P-S6, phosphorylated ribosomal protein s6; p62, sequestosome-1; LC3, microtubule-associated protein light chain 3; P-JNK, phosphorylated c-Jun N-terminal kinase; NF-KB, nuclear factor-κB; TRPC6, transient receptor potential cation channel, subfamily C, member 6; ATP, adenosine triphosphate; TAN, total adenine nucleotides; EC, energy charge; MPTP, mitochondrial permeability transition pore; i.v, intravenous injection; i.p, intraperitoneal injection; i.g, intragastric administration; i.h, hypodermic injection*.

#### Cell Experiments

Sixteen studies were involved in cell experiments. Five studies were published in chinese (Zhu et al., 1999; Guo et al., 2006; Zhao et al., 2012; Cui Q. B. et al., 2013; Xu et al., 2013), and the rest was published in English (Dong et al., 2009; Cui G. et al., 2013; Yin et al., 2013; Fan et al., 2016; Hu et al., 2016; Meng et al., 2016; Wang et al., 2016, 2017; Gao et al., 2017; Zhang X. et al., 2017; Tang et al., 2018) between 1999 and 2017. H9c2 cells (Zhao et al., 2012; Cui G. et al., 2013; Cui Q. B. et al., 2013; Xu et al., 2013; Yin et al., 2013; Hu et al., 2016; Meng et al., 2016; Wang et al., 2016, 2017; Gao et al., 2017; Zhang X. et al., 2017; Tang et al., 2018), neonatal SD rat cardiomyocytes (Dong et al., 2009; Fan et al., 2016), adult wistar rat cardiomyocytes (Zhu et al., 1999), and neonatal wistar rat cardiomyocytes (Guo et al., 2006) were used in 12, 2, 1, and 1 studies/study, respectively. Four kinds of cardiomyocytes injured models were induced by hypoxia/reoxygenation (H/R) (Zhu et al., 1999; Fan et al., 2016; Hu et al., 2016; Meng et al., 2016), 1 by hypoxia (Dong et al., 2009), 1 by ischemia-H/R (Yin et al., 2013), 6 by tert-Butyl hydroperoxide (t-BHP) (Zhao et al., 2012; Cui G. et al., 2013; Cui Q. B. et al., 2013; Xu et al., 2013; Wang et al., 2017; Zhang X. et al., 2017), 2 by Dox (Wang et al., 2016; Tang et al., 2018), 1 by angiotensin II (AngII) (Guo et al., 2006) and 1 by oxygen glucose deprivation (Gao et al., 2017). For outcome measures, 12 studies used the cell viability (Dong et al., 2009; Cui G. et al., 2013; Cui Q. B. et al., 2013; Xu et al., 2013; Yin et al., 2013; Fan et al., 2016; Hu et al., 2016; Wang et al., 2016, 2017; Gao et al., 2017; Zhang X. et al., 2017; Tang et al., 2018), 11 that used apoptosis rate (Guo et al., 2006; Zhao et al., 2012; Cui G. et al., 2013; Yin et al., 2013; Hu et al., 2016; Meng et al., 2016; Wang et al., 2016, 2017; Gao et al., 2017; Zhang X. et al., 2017; Tang et al., 2018), six that used LDH (Dong et al., 2009; Cui G. et al., 2013; Yin et al., 2013; Hu et al., 2016; Wang et al., 2016; Tang et al., 2018). The overall characteristics of included studies are shown in Table 3.

**Table 3:** Characteristics of the 16 included cell studies.

**Study (years)**	**Appellation (*n* = experimental/control group)**	**Organism age tissue**	**Primary cells or subcultured cells**	**Model (method)**	**Treatment group (Method to astragal sides)**	**Control group**	**Outcome Index (time)**	**Intergroup differences**
Zhu et al., 1999	RCM (12/10)	Wistar rats adult myocardium	Primary cells	H/R (40 min/20 min)	Received DSS (40 mg/L) before molding	No treatment	1. Intracellular free calcium concentration	1. *P* < 0.01
Guo et al., 2006	N-RCM (3/3)	Wistar rats neonatal myocardium	Primary cells	Received AngII (1*10^−8^ mol/L)	Received DSS (1*10^−8^ mol/L) for 7 days after molding	No treatment	1. Apoptosis rate	1. *P* < 0.05
Yin et al., 2013	H9c2 (5/5)	BD1X rats embryo myocardium	Subcultured cells	SI/R (2 h/?)	Received DSS (10 um) at the beginning of reperfusion	Received vehicle	1. Cell viability 2. Apoptosis rate 3. LDH 4. Bcl-2/Bax 5. Caspase-3 6. P-Akt 7. P-ERK	1. *P* < 0.01 2. *P* < 0.01 3. *P* < 0.01 4. *P* < 0.01 5. *P* < 0.01 6. *P* < 0.01 7. *P* < 0.05
Meng et al., 2016	H9c2 (5/5)	BD1X rats embryo myocardium	Subcultured cells	H/R (2 h/3 h)	Received DSS (100 mg/l) for 30 min before modeling	No treatment	1. Apoptosis rate 2. P-JNK 3. NF-KB 4. TRPC6 5. Calcium intensity	1. *P* < 0.01 2. *P* < 0.01 3. *P* < 0.01 4. *P* < 0.01 5. *P* < 0.01
Fan et al., 2016	N-RCM (3/3)	SD rats neonatal myocardium	Primary cells	H/R (6 h/18 h)	Received DSS (10 um) for 24 h before modeling	No treatment	1. Cell viability 2. Bcl-2 3. Bax 4. Caspase-3 5. Autophagosomes 6. Autolysosomes 7. P-mTOR 8. P-s6k1 9. P-s6 10. P-62 11. Beclin-1 12. LC3-II	1. *P* < 0.01 2. *P* < 0.01 3. *P* < 0.01 4. *P* < 0.01 5. *P* < 0.01 6. *P* < 0.01 7. *P* < 0.01 8. *P* < 0.01 9. *P* < 0.01 10. *P* < 0.01 11. *P* < 0.01 12. *P* < 0.01
Hu et al., 2016	H9c2 (6/6)	BD1X rats embryo myocardium	Subcultured cells	H/R (4 h/20 h)	Received DSS (80 um) at the beginning of re-oxygenation	No treatment	1. Cell viability 2. Apoptosis rate 3. LDH 4. Bcl-2/Bax 5. Caspase-3 6. P-Akt 7. Nrf2 8. HO-1	1. *P* < 0.05 2. *P* < 0.01 3. *P* < 0.05 4. *P* < 0.01 5. *P* < 0.01 6. *P* < 0.01 7. *P* < 0.01 8. *P* < 0.05
Gao et al., 2017	H9c2 (3/3)	BD1X rats embryo myocardium	Subcultured cells	Oxygen glucose deprivation model	Received DSS (10 um) at the beginning of re-oxygenation	No treatment	1. Cell viability 2. Apoptosis rate 3. ATP 5g1 mRNA 4. MPTP	1. *P* < 0.05 2. *P* < 0.01 3. *P* < 0.05 4. *P* < 0.05
Dong et al., 2009	N-RCM (6/6)	SD rats neonatal myocardium	Primary cells	Hypoxia for 5 h.	Received DSS (1 um) for 12 h before modeling	No treatment	1. Cell viability 2. MDA 3. LDH 4. Bcl-2 5. Bax 6. Caspase-3	1. *P* < 0.05 2. *P* < 0.05 3. *P* < 0.05 4. *P* < 0.05 5. *P* < 0.05 6. *P* < 0.05
Zhao et al., 2012	H9c2 (4/4)	BD1X rats embryo myocardium	Subcultured cells	Received t-BHP (150 um) for 12 h	Received DSS (400 um) for 1 h before modeling	No treatment	1. Apoptosis rate	1. *P* < 0.01
Cui G. et al., 2013	H9c2 (3/3)	BD1X rats embryo myocardium	Subcultured cells	Received t-BHP (150 um) for 2.5 h	Received DSS (300 um) for 2.5 h before modeling	No treatment	1. Cell viability 2. Apoptosis rate 3. LDH 4. Δψm 5. Intracellular oxidant level 6. P-AKT 7. P-PI3K 8. P-GS3K-3β	1. *P* < 0.001 2. *P* < 0.001 3. *P* < 0.001 4. *P* < 0.05 5. *P* < 0.01 6. *P* < 0.05 7. *P* < 0.05 8. *P* < 0.05
Cui Q. B. et al., 2013	H9c2 (3/3)	BD1X rats embryo myocardium	Subcultured cells	Received t-BHP (150 um) for 12 h	Received DSS (250 um) for 1 h before modeling	No treatment	1. Cell viability	1. *P* < 0.05
Xu et al., 2013	H9c2 (3/3)	BD1X rats embryo myocardium	Subcultured cells	Received t-BHP (150 um) for 12 h	Received DSS (250 um) for 1 h before modeling	No treatment	1. Cell viability	1. *P* < 0.01
Wang et al., 2016	H9c2 (3/3)	BD1X rats embryo myocardium	Subcultured cells	Received DOX (1 um)	Received DSS (10 um) for 24 h after modeling	No treatment	1. Cell viability 2. Apoptosis rate 3. LDH 4. ATP-levels 5. NRF-1 6. PGC-1α 7. Nrf2 8. Caspase-3 9. mtDNA	1. *P* < 0.05 2. *P* < 0.05 3. *P* < 0.05 4. *P* < 0.05 5. *P* < 0.05 6. *P* < 0.05 7. *P* < 0.05 8. *P* < 0.05 9. *P* < 0.05
Zhang X. et al., 2017	H9c2 (3/3)	BD1X rats embryo myocardium	Subcultured cells	Received t-BHP (150 um/l) for 4 h	Received DSS (100 um) for 1 h before modeling	No treatment	1. Cell viability 2. Apoptotic cells 3. OH– 4. O2– 5. ONOO– 6. Bcl-2/Bax 7. Caspase 3 8. PGC-1α 9. Nrf2 10. Tfam 11. HO-1	1. *P* < 0.001 2. *P* < 0.001 3. *P* < 0.001 4. *P* < 0.001 5. *P* < 0.001 6. *P* < 0.001 7. *P* < 0.05 8. *P* < 0.01 9. *P* < 0.05 10. *P* < 0.05 11. *P* < 0.05
Wang et al., 2017	H9c2 (3/3)	BD1X rats embryo myocardium	Subcultured cells	Received t-BHP (150 um) for 3 h	Received DSS (100 um) for 1 h before modeling	No treatment	1. Cell viability 2. Apoptotic cells 3. Bcl-2/Bax 4. Caspase 3 5. Cytochrome C	1. *P* < 0.01 2. *P* < 0.01 3. *P* < 0.01 4. *P* < 0.05 5. *P* < 0.05
Tang et al., 2018	H9c2 (3/3)	BD1X rats embryo myocardium	Subcultured cells	Received DOX (1 um)	Received DSS (20 um) for 24 h after modeling	No treatment	1. Cell viability 2. LDH 3. P-ERK 4. P-JNK 5. Apotosis 6. Caspase-3 7. Caspase-7 8. LC3II/LC3I	1. *P* < 0.0001 2. *P* < 0.0001 3. *P* < 0.01 4. *P* < 0.01 5. *P* < 0.0001 6. *P* < 0.01 7. *P* < 0.0001 8. *P* < 0.01

*RCM, rat cardiac myocytes, N-RCM, neonatal rat cardiac myocytes, BD1X rats, rattus norvegicus rats, H/R, hypoxia/reoxygenation, SI/R, simulated ischemia-reperfusion; t-BHP, tert-Butyl hydroperoxide; DOX, doxorubicin; DSS, Danshensu; LDH, lactate dehydrogenase; P-GSK-3β, phosphorylated Glycogen synthase kinase-3 beta; P-Akt, phosphothreonine kinase; p-ERK, phosphorylated extracellular regulated protein kinases; PGC1-α, peroxisome proliferator activated receptor γ coactivator-1α; Nrf2, nuclear factor erythroid 2-related factor 2; HO-1, heme oxygenase-1; MDA, malondialdehyde; Bcl-2, B-cell lymphoma-2; Bax, BCL2-associated X protein; P-mTOR, phosphorylated mammalian target of rapamycin; P-S6k1, phosphorylated ribosomal protein S6 kinase beta-1; P-S6, phosphorylated ribosomal protein s6; p62, sequestosome-1; LC3, microtubule-associated protein light chain 3; P-JNK, phosphorylated c-Jun N-terminal kinase; NF-KB, nuclear factor-κB; TRPC6, transient receptor potential cation channel, subfamily C, member 6; MPTP, mitochondrial permeability transition pore; Δψm, mitochondrial membrane potential; mtDNA, mitochondrial DNA; Tfam, mitochondrial transcription factor A; NRF-1, nuclear respiratory factor 1*.

### Study Quality

#### Animal Studies

The quality score of included studies ranged from 3 to 7. All studies obtained two points from peer-reviewed publication, and anesthetic without significant cardioprotective (Jiang et al., 1982; Tang et al., 1989, 2018; Li et al., 1996, 2012, 2016; Dong et al., 2009; Xiang et al., 2009; Cheng et al., 2010; Lu et al., 2010; Zhang et al., 2010; Quan et al., 2012; Zhao et al., 2012; Cui G. et al., 2013; Yin et al., 2013, 2017; Chen et al., 2014; Cui et al., 2014; Yu J. et al., 2015; Fan et al., 2016; Hu et al., 2016; Song et al., 2016; Wang et al., 2016, 2017; Wei et al., 2016; Gao et al., 2017; Zhang X. et al., 2017). Twenty-three studies reported random allocation (Jiang et al., 1982; Dong et al., 2009; Xiang et al., 2009; Cheng et al., 2010; Lu et al., 2010; Zhang et al., 2010; Li et al., 2012, 2016; Quan et al., 2012; Zhao et al., 2012; Cui G. et al., 2013; Yin et al., 2013, 2017; Chen et al., 2014; Cui et al., 2014; Yu J. et al., 2015; Fan et al., 2016; Hu et al., 2016; Song et al., 2016; Wei et al., 2016; Gao et al., 2017; Wang et al., 2017; Zhang X. et al., 2017). None of studies mentioned blinded assessment of outcome, described a sample size calculation, or used appropriate animal model. One study used a blind method in modeling (Yin et al., 2017). Twenty-four studies reported the control of temperature (Tang et al., 1989, 2018; Li et al., 1996, 2012, 2016; Dong et al., 2009; Xiang et al., 2009; Cheng et al., 2010; Zhang et al., 2010; Quan et al., 2012; Zhao et al., 2012; Cui G. et al., 2013; Yin et al., 2013, 2017; Chen et al., 2014; Cui et al., 2014; Yu J. et al., 2015; Fan et al., 2016; Hu et al., 2016; Song et al., 2016; Wang et al., 2016, 2017; Wei et al., 2016; Gao et al., 2017), 13 studies complianced with animal welfare regulations (Li et al., 2012, 2016; Cui G. et al., 2013; Yin et al., 2013, 2017; Yu J. et al., 2015; Fan et al., 2016; Hu et al., 2016; Song et al., 2016; Wang et al., 2016, 2017; Gao et al., 2017; Tang et al., 2018) and 17 studies declared the potential conflict of interests (Dong et al., 2009; Zhang et al., 2010; Li et al., 2012, 2016; Cui G. et al., 2013; Yin et al., 2013, 2017; Cui et al., 2014; Yu J. et al., 2015; Fan et al., 2016; Hu et al., 2016; Song et al., 2016; Wang et al., 2016, 2017; Gao et al., 2017; Zhang X. et al., 2017; Tang et al., 2018). The methodological quality of each study was summarized in Table 4.

**Table 4:** Risk of bias of the included *in vivo* studies.

**Study**	**A**	**B**	**C**	**D**	**E**	**F**	**G**	**H**	**I**	**J**	**Total**
Jiang et al., 1982	**√**		**√**			**√**					3
Tang et al., 1989	**√**	**√**				**√**					3
Li et al., 1996	**√**	**√**				**√**					3
Zhang et al., 2010	**√**	**√**	**√**			**√**				**√**	5
Lu et al., 2010	**√**		**√**			**√**					3
Li et al., 2012	**√**	**√**	**√**			**√**			**√**	**√**	6
Yin et al., 2013	**√**	**√**	**√**			**√**			**√**	**√**	6
Yu J. et al., 2015	**√**	**√**	**√**			**√**			**√**	**√**	6
Wei et al., 2016	**√**	**√**	**√**			**√**					4
Song et al., 2016	**√**	**√**	**√**			**√**			**√**	**√**	6
Fan et al., 2016	**√**	**√**	**√**			**√**			**√**	**√**	6
Li et al., 2016	**√**	**√**	**√**			**√**			**√**	**√**	6
Hu et al., 2016	**√**	**√**	**√**			**√**			**√**	**√**	6
Yin et al., 2017	**√**	**√**	**√**	**√**		**√**			**√**	**√**	7
Gao et al., 2017	**√**	**√**	**√**			**√**			**√**	**√**	6
Dong et al., 2009	**√**	**√**	**√**			**√**				**√**	5
Xiang et al., 2009	**√**	**√**	**√**			**√**					4
Cheng et al., 2010	**√**	**√**	**√**			**√**					4
Zhao et al., 2012	**√**	**√**	**√**			**√**					4
Quan et al., 2012	**√**	**√**	**√**			**√**					4
Cui G. et al., 2013	**√**	**√**	**√**			**√**			**√**	**√**	6
Chen et al., 2014	**√**	**√**	**√**			**√**					4
Cui et al., 2014	**√**	**√**	**√**			**√**				**√**	5
Wang et al., 2016	**√**	**√**				**√**			**√**	**√**	5
Zhang X. et al., 2017	**√**		**√**			**√**				**√**	4
Wang et al., 2017	**√**	**√**	**√**			**√**			**√**	**√**	6
Tang et al., 2018	**√**	**√**				**√**			**√**	**√**	5

*Studies fulfilling the criteria of: A, peer reviewed publication; B, control of temperature; C, random allocation to treatment or control; D, blinded induction of model; E, blinded assessment of outcome; F, use of anesthetic without significant intrinsic vascular protection activity; G, appropriate animal model (aged, diabetic, or hypertensive); H, sample size calculation; I, compliance with animal welfare regulations; J, statement of potential conflict of interests*.

#### Cell Studies

The quality score of included studies ranged from 3 to 6. All the studies obtained two points from peer-reviewed publication, and culture environment (Zhu et al., 1999; Guo et al., 2006; Dong et al., 2009; Zhao et al., 2012; Cui G. et al., 2013; Cui Q. B. et al., 2013; Xu et al., 2013; Yin et al., 2013; Fan et al., 2016; Hu et al., 2016; Meng et al., 2016; Wang et al., 2016, 2017; Gao et al., 2017; Zhang X. et al., 2017; Tang et al., 2018). No study described a sample size calculation. One study used use primary adult cardiomyocytes (Zhu et al., 1999), three studies exameid the toxicity of treatment on cells (Cui G. et al., 2013; Wang et al., 2016; Tang et al., 2018) and four stuides mentioned random allocation to treatment or control (Zhao et al., 2012; Xu et al., 2013; Yin et al., 2013; Hu et al., 2016). Seven studies reported cell lines with reliable source or validated by appropriate methods (Guo et al., 2006; Dong et al., 2009; Cui G. et al., 2013; Yin et al., 2013; Fan et al., 2016; Hu et al., 2016; Meng et al., 2016). Twelve studies declared the potential conflict of interests (Dong et al., 2009; Cui G. et al., 2013; Cui Q. B. et al., 2013; Yin et al., 2013; Fan et al., 2016; Hu et al., 2016; Meng et al., 2016; Wang et al., 2016, 2017; Gao et al., 2017; Zhang X. et al., 2017; Tang et al., 2018). The methodological quality of each study was summarized in Table 5.

**Table 5:** Risk of bias of the included *in vitro* studies.

**Study**	**A**	**B**	**C**	**D**	**E**	**F**	**G**	**H**	**I**	**J**	**Total**
Zhu et al., 1999	**√**	**√**			**√**						3
Guo et al., 2006	**√**		**√**		**√**						3
Yin et al., 2013	**√**		**√**		**√**	**√**	**√**			**√**	6
Meng et al., 2016	**√**		**√**		**√**					**√**	4
Fan et al., 2016	**√**		**√**		**√**					**√**	4
Hu et al., 2016	**√**		**√**		**√**	**√**				**√**	5
Gao et al., 2017	**√**				**√**					**√**	3
Dong et al., 2009	**√**		**√**		**√**					**√**	4
Zhao et al., 2012	**√**				**√**	**√**					3
Cui G. et al., 2013	**√**			**√**	**√**					**√**	4
Cui G. et al., 2013	**√**		**√**	**√**	**√**	**√**				**√**	4
Xu et al., 2013	**√**				**√**					**√**	3
Wang et al., 2016	**√**				**√**					**√**	4
Zhang X. et al., 2017	**√**				**√**					**√**	3
Wang et al., 2017	**√**				**√**						3
Tang et al., 2018	**√**			**√**	**√**					**√**	4

*Studies fulfilling the criteria of: A, peer reviewed publication; B, use primary adult cardiomyocytes to study; C, cell lines with reliable source or validated by appropriate methods; D, assess toxicity of treatment on cells; E, culture environment (culture media/sera, pH/CO_2_, and temperature); F, random allocation to treatment or control; G, blinded induction of model; H, blinded assessment of outcome; I, calculation of the sample size necessary to achieve sufficient power; J, statement of potential conflict of interest*.

### Effectiveness

#### MI Size

**Figure 2:**
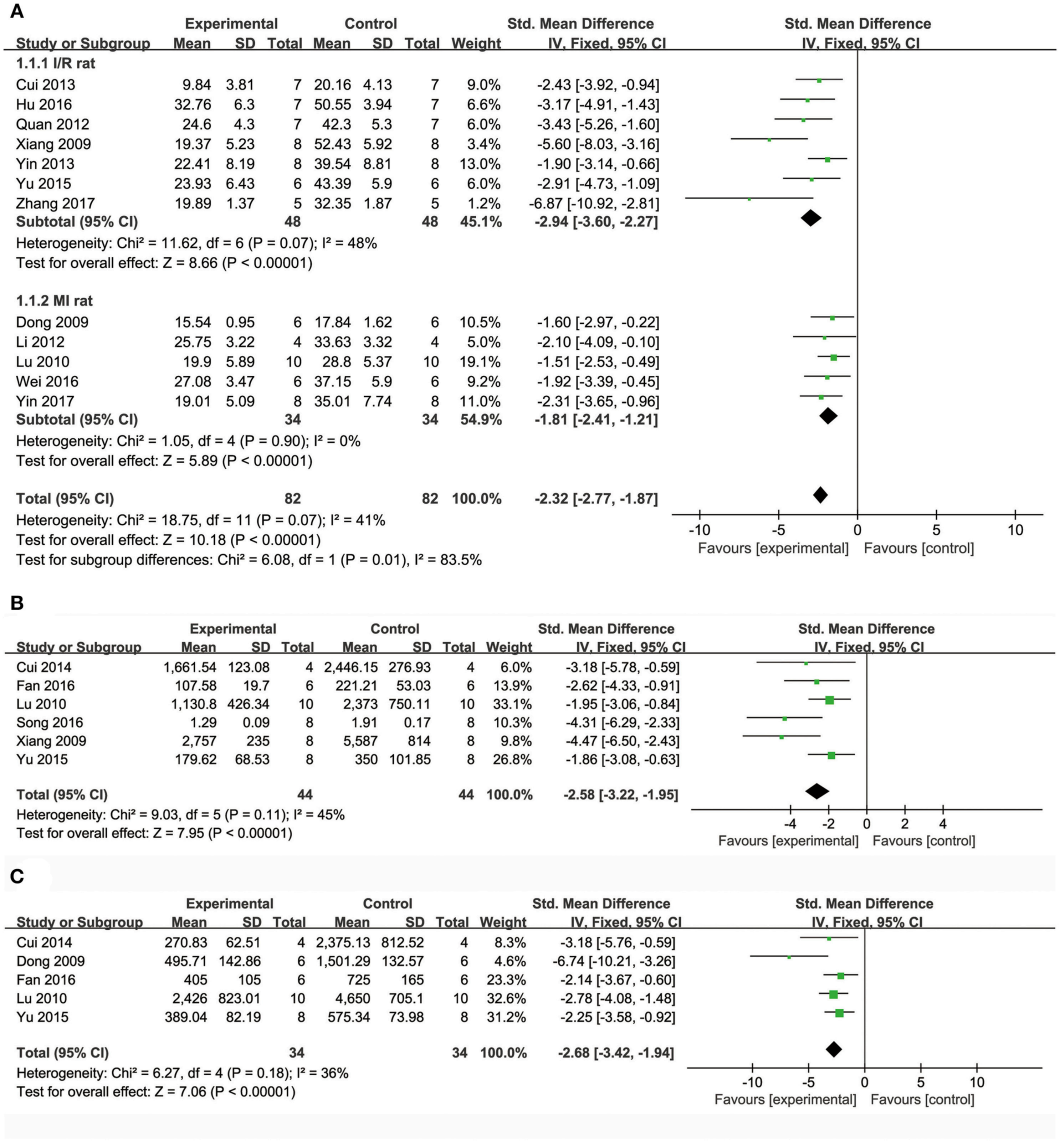
The forest plot: **(A)** *in vivo* effects of danshensu on reducing myocardial infarction size compared with controls (*n* = 82 per group); **(B)** *in vivo* effects of danshensu on reducing LDH compared with controls (*n* = 44 per group); **(C)** *in vivo* effects of danshensu on reducing CK compared with controls (*n* = 34per group). LDH, lactate dehydrogenase; CK, creatine kinase.

Meta-analysis of 15 studies (Dong et al., 2009; Xiang et al., 2009; Lu et al., 2010; Li et al., 2012; Quan et al., 2012; Cui G. et al., 2013; Yin et al., 2013, 2017; Chen et al., 2014; Cui et al., 2014; Yu J. et al., 2015; Hu et al., 2016; Wei et al., 2016; Wang et al., 2017; Zhang X. et al., 2017) showed DSS has significant effects on reducing MI size compared with control (*n* = 231, SMD: −2.27, 95% CI: −2.65 to −1.89, *P* < 0.00001; heterogeneity χ^2^ = 52.89, *P* < 0.00001, *I*^2^ = 74%). We conducted subgroup analysis according to the different animal models because of high heterogeneity. Meta-analysis of seven studies (Xiang et al., 2009; Quan et al., 2012; Cui G. et al., 2013; Yin et al., 2013; Yu J. et al., 2015; Hu et al., 2016; Zhang X. et al., 2017) showed more significant effect than that of controls according to rat models of I/R (*n* = 96, SMD: −2.94, 95% CI: −3.60 to −2.27, *P* < 0.00001; heterogeneity χ^2^ = 11.62, *P* = 0.07, *I*^2^ = 48%; Figure 2A). Meta-analysis of five studies (Dong et al., 2009; Lu et al., 2010; Li et al., 2012; Wei et al., 2016; Yin et al., 2017) showed significant effects of DSS for reducing MI size than that of controls according to rat models of MI (*n* = 68, SMD: −1.81, 95% CI: −2.41 to −1.21, *P* < 0.00001; heterogeneity χ^2^ = 1.05, *P* = 0.90, *I*^2^ = 0%; Figure 2A). Moreover, one study (Jiang et al., 1982) showed positive effect of DSS on reducing MI size in canines (*P* < 0.05).

#### Biochemical Markers of MI

Meta-analysis of 8 studies (Dong et al., 2009; Xiang et al., 2009; Lu et al., 2010; Li et al., 2012; Cui et al., 2014; Yu J. et al., 2015; Fan et al., 2016; Song et al., 2016) showed DSS has significant effects on reducing LDH compared with controls (*n* = 116, SMD: −2.81, 95% CI: −3.44 to −2.18, *P* < 0.00001; heterogeneity χ^2^ = 35.07, *P* < 0.0001, *I*^2^ = 80%). Two studies (Dong et al., 2009; Li et al., 2012) were removed through sensitivity analysis for simultaneous application of pretreatment and post-injury treatment. The remain six studies (Xiang et al., 2009; Lu et al., 2010; Cui et al., 2014; Yu J. et al., 2015; Fan et al., 2016; Song et al., 2016) showed significant effects on decreasing LDH in analysis (*n* = 88, SMD: −2.58, 95% CI: −3.22 to −1.95, *P* < 0.00001; heterogeneity χ^2^ = 9.03, *P* = 0.11, *I*^2^ = 45%; Figure 2B). Meta-analysis of seven studies (Dong et al., 2009; Xiang et al., 2009; Lu et al., 2010; Cui et al., 2014; Yu J. et al., 2015; Fan et al., 2016; Song et al., 2016) showed DSS has significant effects on decreasing CK compared with controls (*n* = 100, SMD: −3.16, 95% CI: −3.86 to −2.46, *P* < 0.00001; heterogeneity χ^2^ = 20.32, *P* = 0.002, *I*^2^ = 70%). Through sensitivity analysis, we removed two studies which utilized ISO in modeling (Song et al., 2016) and the other with unknown administration route (Xiang et al., 2009). Meta-analysis of 5 studies (Dong et al., 2009; Lu et al., 2010; Cui et al., 2014; Yu J. et al., 2015; Fan et al., 2016) showed the significant effects on reducing CK (*n* = 68, SMD: −2.68, 95% CI: −3.42 to −1.94, *P* < 0.00001; heterogeneity χ^2^ = 6.27, *P* = 0.18, *I*^2^ = 36%; Figure 2C). Five studies (Li et al., 2012, 2016; Quan et al., 2012; Yin et al., 2013; Hu et al., 2016) failed to pool the analysis, but they all showed DSS has significant effects on decreasing CK-MB compared with control (*P* < 0.05, or *P* < 0.01, or *P* < 0.001). One study (Li et al., 2016) showed DSS has significant effects on decreasing cTnT (*P* < 0.001).

##### The level of ST-segment elevation and/or LVEFand/or LVFS

One study (Li et al., 2012) showed DSS significantly inhibit the ST-segment elevation compared with control (*P* < 0.001). Anothe study (Yin et al., 2017) showed positive effects of DSS on improving LVEF and LVFS compared with control (*P* < 0.05).

##### Cardioprotective mechanisms

**Figure 3:**
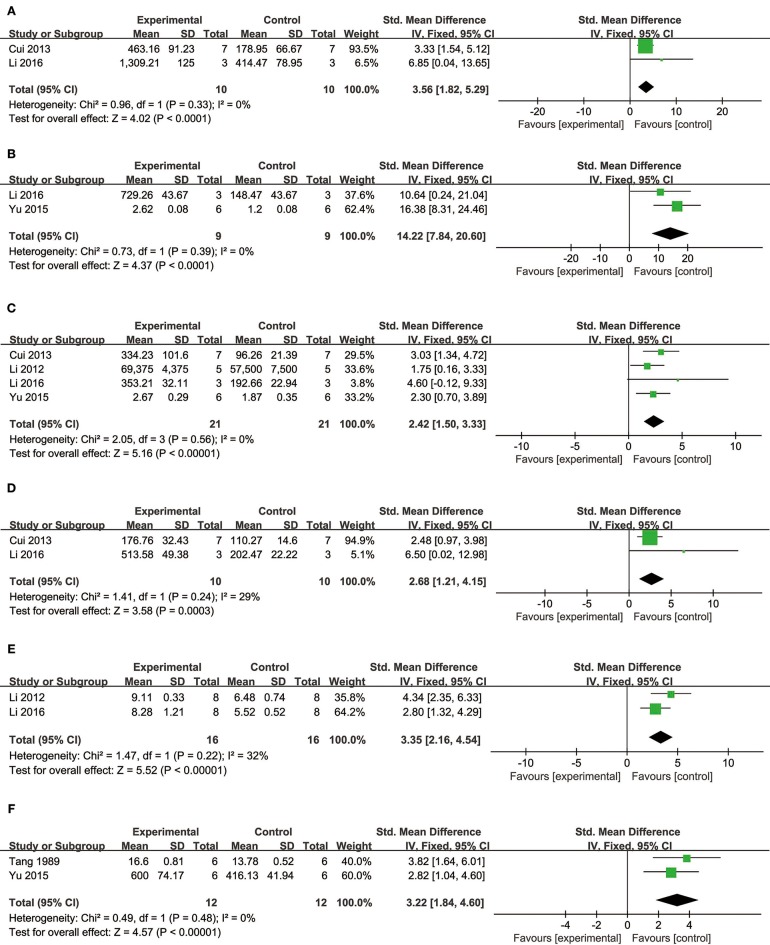
The forest plot: **(A)** *in vivo* effects of danshensu for increasing p-PI3K compared with controls (*n* = 10 per group); **(B)** *in vivo* effects of danshensu for increasing p-AKT compared with controls (*n* = 9 per group); **(C)** *in vivo* effects of danshensu for increasing Nrf2 compared with controls (*n* = 21per group); **(D)** *in vivo* effects of danshensu for increasing HO-1compared with controls (*n* = 10 per group); **(E)** *in vivo* effects of danshensu for increasing GST compared with controls (*n* = 16 per group); **(F)** *in vivo* effects of danshensu for increasing GSH-Pxcompared with controls (*n* = 12 per group). p-PI3K, phosphatidylinositol 3-kinase; p-AKT, phosphothreonine kinase; Nrf2, nuclear factor erythroid 2-related factor 2; HO-1, heme oxygenase-1; GST, glutathione-S-transferase; GSH-Px, glutathione peroxidase.

**Figure 4:**
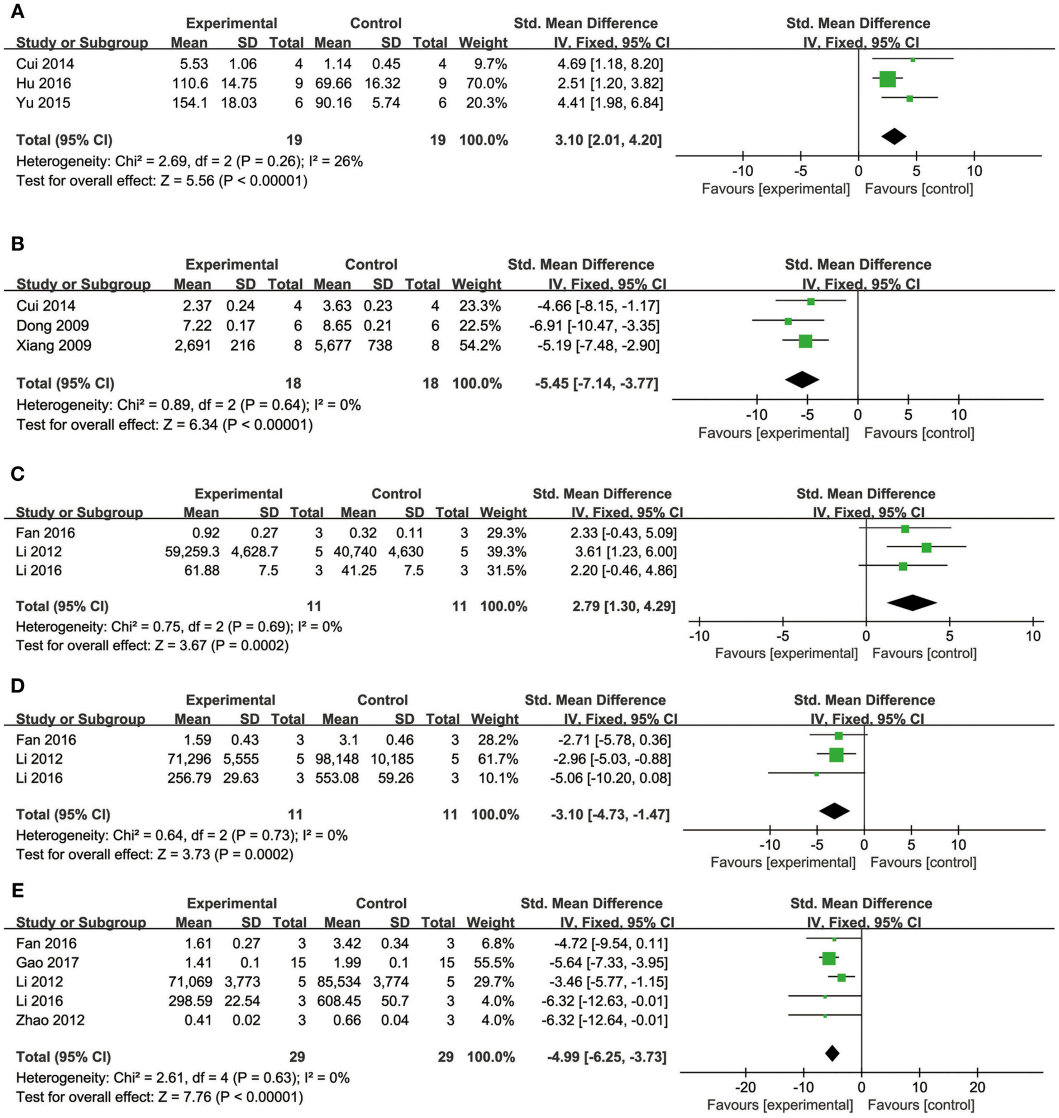
The forest plot: **(A)** *in vivo* effects of danshensu for increasing SOD compared with controls (*n* = 19 per group); **(B)** *in vivo* effects of danshensu for reducing MDA compared with controls (*n* = 18 per group); **(C)** *in vivo* effects of danshensu for increasing Bcl-2 compared with controls (*n* = 11 per group); **(D)** *in vivo* effects of danshensu for reducing Bax compared with controls (*n* = 11 per group); **(E)** *in vivo* effects of danshensu for reducing caspase-3 compared with controls (*n* = 29 per group). SOD, superoxide dismutase; MDA, malondialdehyde; Bcl-2, B-cell lymphoma-2; Bax, BCL2-associated X protein.

**Figure 5:**
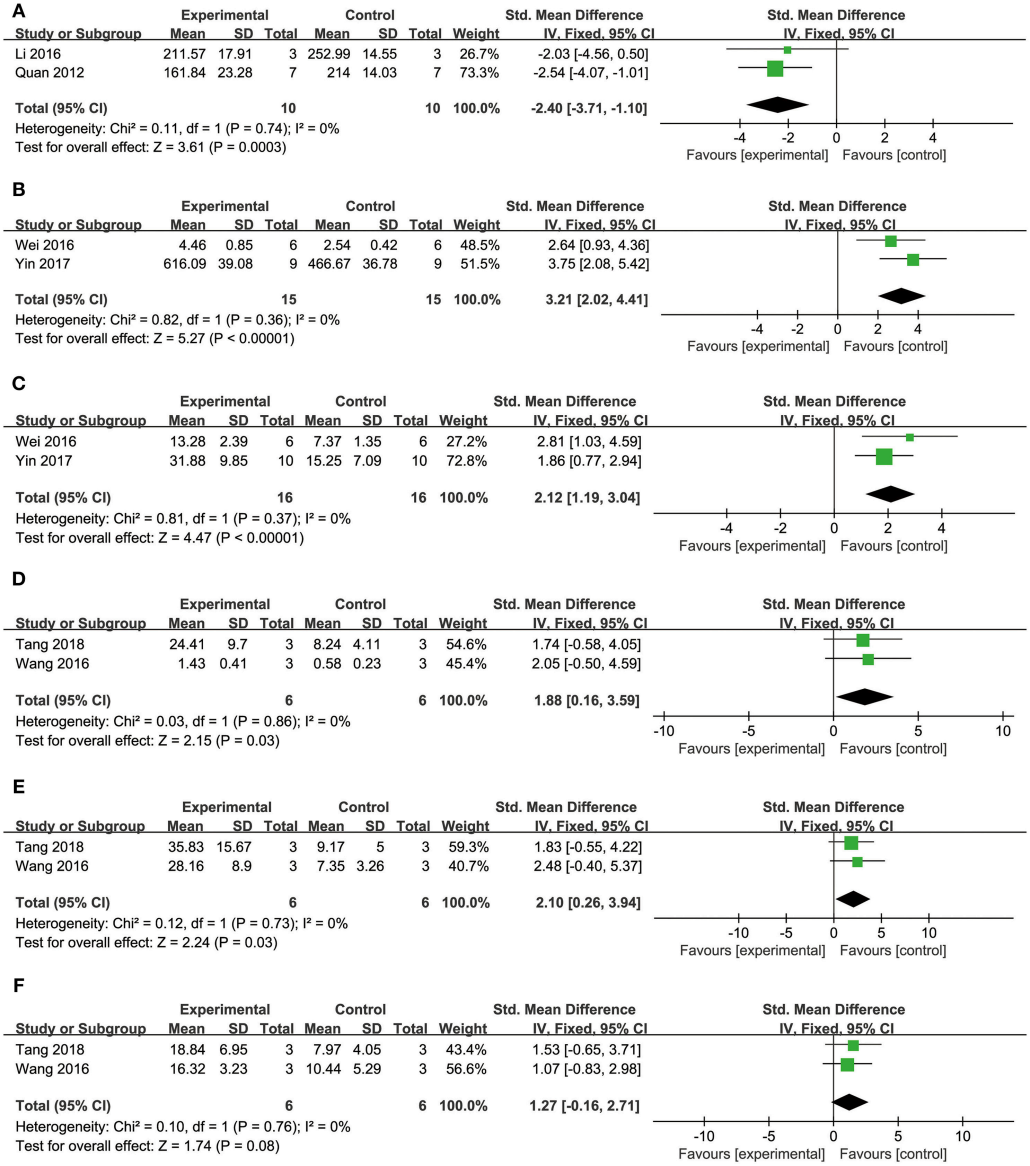
The forest plot: **(A)** *in vivo* effects of danshensu for reducing TNF-α compared with controls (*n* = 10 per group); **(B)** *in vivo* effects of danshensu for increasing SDF-1 compared with controls (*n* = 15 per group); **(C)** *in vivo* effects of danshensu for increasing MVD compared with controls (*n* = 16 per group); **(D)** *in vivo* effects of danshensu for increasing stroke volume compared with controls (*n* = 6 per group); **(E)** *in vivo* effects of danshensu for increasing cardiac output compared with controls (*n* = 6 per group); **(F)** *in vivo* effects of danshensu for increasing fractional shortening compared with controls (*n* = 6 per group). TNF-α, tumor necrosis factor-α; SDF-1, stromal cell-derived factor-1 α; MVD, micro vessel density.

Comparing DSS with controls, meta-analysis of two studies (Cui G. et al., 2013; Li et al., 2016) showed significant effects on increasing phosphatidylinositol 3-kinase (p-PI3K) (*n* = 20, SMD: 3.56, 95% CI: 1.82–5.29, *P* < 0.0001; heterogeneity χ^2^ = 0.96, *P* = 0.33, *I*^2^ = 0%; Figure 3A); two studies (Yu J. et al., 2015; Li et al., 2016) for increasing phosphothreonine kinase (p-Akt) (*n* = 18, SMD: 14.22, 95% CI: 7.84–20.60, *P* < 0.0001; heterogeneity χ^2^ = 0.73, *P* = 0.39, *I*^2^ = 0%; Figure 3B); four studies (Li et al., 2012, 2016; Cui G. et al., 2013; Yu J. et al., 2015) for increasing nuclear factor erythroid 2-related factor 2 (Nrf2) (*n* = 42, SMD: 2.42, 95% CI: 1.50–3.33, *P* < 0.00001; heterogeneity χ^2^ = 2.05, *P* = 0.56, *I*^2^ = 0%; Figure 3C); 2 studies (Cui G. et al., 2013; Li et al., 2016) for increasing heme oxygenase-1 (HO-1) (*n* = 20, SMD: 2.68, 95% CI: 1.21–4.15, *P* = 0.0003; heterogeneity χ^2^ = 1.41, *P* = 0.24, *I*^2^ = 29%; Figure 3D); two studies (Li et al., 2012, 2016) for increasing glutathione-S-transferase (GST) (*n* = 32, SMD: 3.35, 95% CI: 2.16–4.54, *P* < 0.00001; heterogeneity χ^2^ = 1.47, *P* = 0.22, *I*^2^ = 32%; Figure 3E); two studies (Tang et al., 1989; Yu J. et al., 2015) for increasing Glutathione peroxidase (GSH-Px) (*n* = 24, SMD: 3.22, 95% CI: 1.84–4.60, *P* < 0.00001; heterogeneity χ^2^ = 0.49, *P* = 0.48, *I*^2^ = 0%; Figure 3F); two studies (Li et al., 2012, 2016) for increasing glutathione synthetase (GSH) (*P* < 0.001); three studies (Cui et al., 2014; Yu J. et al., 2015; Hu et al., 2016) for increasing superoxide dismutase (SOD) (*n* = 38, SMD: 3.10, 95% CI: 2.10–4.20, *P* < 0.00001; heterogeneity χ^2^ = 2.69, *P* = 0.26, *I*^2^ = 26%; Figure 4A); three studies(Dong et al., 2009; Xiang et al., 2009; Cui et al., 2014) for reducing malondialdehyde (MDA) (*n* = 36, SMD: −5.45, 95% CI: −7.14 to −3.77, *P* < 0.00001; heterogeneity χ^2^ = 0.89, *P* = 0.64, *I*^2^ = 0%; Figure 4B); three studies (Li et al., 2012, 2016; Fan et al., 2016) for increasing B-cell lymphoma-2 (Bcl-2) (*n* = 22, SMD: 2.79, 95% CI: 1.30–4.29, *P* = 0.0002; heterogeneity χ^2^ = 0.75, *P* = 0.69, *I*^2^ = 0%; Figure 4C); three studies (Li et al., 2012, 2016; Fan et al., 2016) for reducing BCL2-associated X protein (Bax) (*n* = 22, SMD: −3.10, 95% CI: −4.73 to −1.47, *P* = 0.0002; heterogeneity χ^2^ = 0.64, *P* = 0.73, *I*^2^ = 0%; Figure 4D); one study (Zhao et al., 2012) for reducing Bcl2/Bax (*P* < 0.01); five studies (Li et al., 2012, 2016; Zhao et al., 2012; Fan et al., 2016; Gao et al., 2017) for reducing caspase-3 (*n* = 58, SMD: −4.99, 95% CI: −6.25 to −3.73, *P* < 0.00001; heterogeneity χ^2^ = 2.61, *P* = 0.63, *I*^2^ = 0%; Figure 4E); two studies (Quan et al., 2012; Li et al., 2016) for reducing tumor necrosis factor-α (TNF-α) (*n* = 20, SMD: −2.40, 95% CI: −3.71 to −1.10, *P* = 0.0003; heterogeneity χ^2^ = 0.11, *P* = 0.74, *I*^2^ = 0%; Figure 5A); one study (Quan et al., 2012) for reducing Interleukin-1 (IL-1) and Interleukin-6 (IL-6) (*P* < 0.05); two studies (Wei et al., 2016; Yin et al., 2017) for increasing stromal cell-derived factor-1α (SDF-1) (*n* = 30, SMD: 3.21, 95% CI: 2.02–4.41, *P* < 0.00001; heterogeneity χ^2^ = 0.82, *P* = 0.36, *I*^2^ = 0%; Figure 5B), micro vessel density (MVD) (*n* = 32, SMD: 2.12, 95% CI: 1.19–3.04, *P* < 0.00001; heterogeneity χ^2^ = 0.81, *P* = 0.37, *I*^2^ = 0%; Figure 5C), C-X-C chemokine receptor type 4 (CXCR-4) (*P* < 0.01) and vascular endothelial growth factor (VEGF) (*P* < 0.05); one study (Yin et al., 2017) for increasing basic fibroblast growth factor (bFGF) (*P* < 0.05); three studies (Li et al., 1996; Cheng et al., 2010; Zhang et al., 2010) for increasing adenosine triphosphate (ATP) (*P* < 0.01 ORP < 0.05 ORP < 0.001); two studies (Li et al., 1996; Zhang et al., 2010) for increasingenergy charge(EC) (*P* < 0.01); one study (Gao et al., 2017) for inhibiting Mitochondrial permeability transition pore (MPTP) opening (*P* < 0.05); one study (Fan et al., 2016) for increasing phosphorylated mammalian target of rapamycin (p-mTOR) (*P* < 0.05), two studies (Wang et al., 2016; Tang et al., 2018) for increasing stroke volum (*n* = 12, SMD: 1.88, 95% CI: 0.16–3.59, *P* = 0.03; heterogeneity χ^2^ = 0.03, *P* = 0.86, *I*^2^ = 0%; Figure 5D), cardiac output (*n* = 12, SMD: 2.10, 95% CI: 0.26–3.94, *P* = 0.03; heterogeneity χ^2^ = 0.12, *P* = 0.73, *I*^2^ = 0%; Figure 5E) and fractional shortening (*n* = 12, SMD: 1.27, 95% CI: −0.16 to 2.71, *P* = 0.08; heterogeneity χ^2^ = 0.10, *P* = 0.76, *I*^2^ = 0%; Figure 5F). We summarized a schematic representation of cardioprotective mechanism of DSS for myocardial ischemic injury (**Figure 8**).

#### Cell Viability and Apoptosis Rate

##### Cell viability

**Figure 6:**
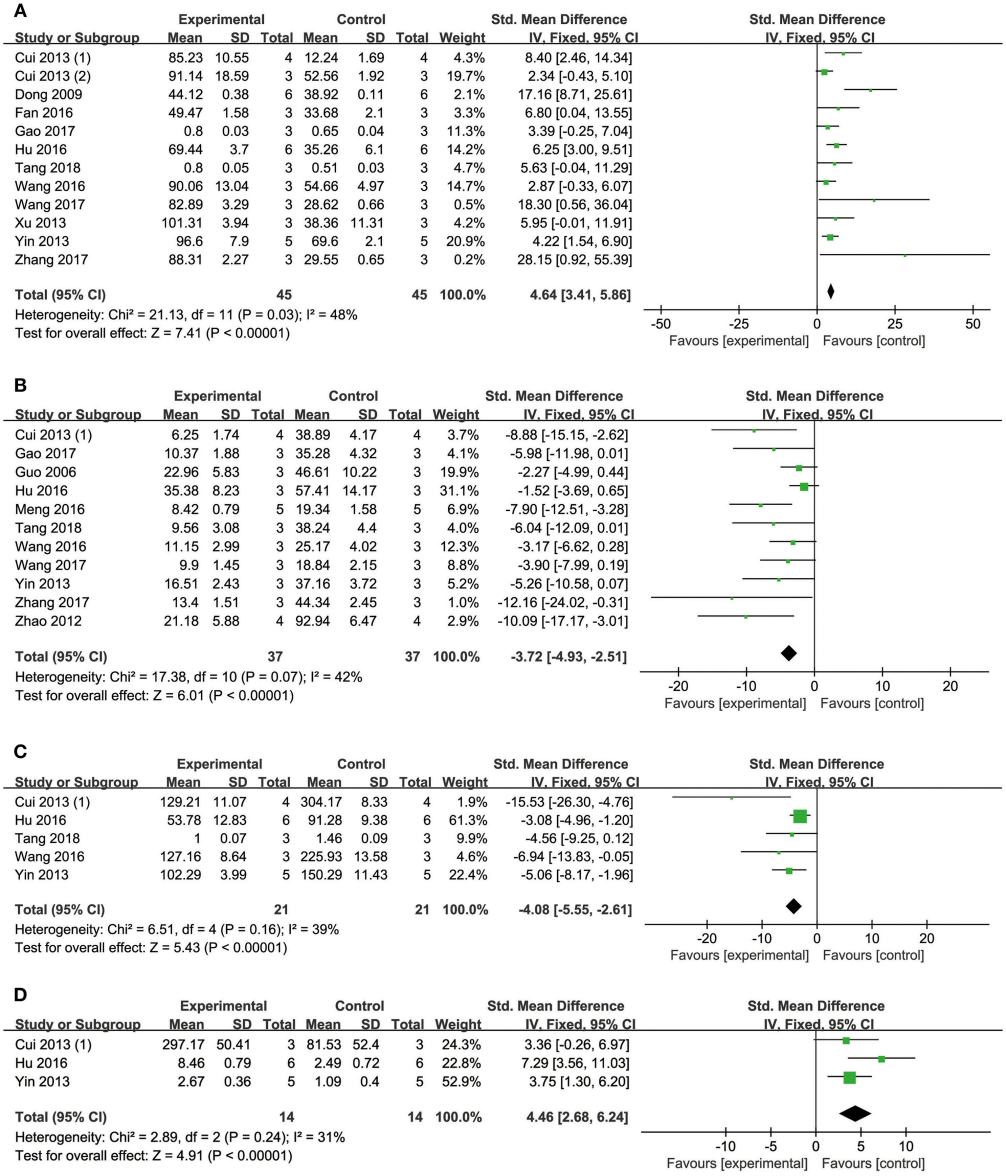
The forest plot: **(A)** *in vitro* effects of danshensu for increasing cell viability compared with controls (*n* = 45 per group); **(B)** *in vitro* effects of danshensu for reducing apoptosis rate compared with controls (*n* = 37 per group); **(C)** *in vitro* effects of danshensu for reducing LDH compared with controls (*n* = 21 per group); (**D)** *in vitro* effects of danshensu for increasing p-AKT compared with controls (*n* = 14 per group). LDH, lactate dehydrogenase; p-AKT, phosphothreonine kinase.

Meta-analysis of 12 studies (Dong et al., 2009; Cui G. et al., 2013; Cui Q. B. et al., 2013; Xu et al., 2013; Yin et al., 2013; Fan et al., 2016; Hu et al., 2016; Wang et al., 2016, 2017; Gao et al., 2017; Zhang X. et al., 2017; Tang et al., 2018) showed DSS has significant effects on improving cell viability compared with controls (*n* = 90, SMD: 4.64, 95% CI: 3.41–5.86, *P* < 0.00001; heterogeneity χ^2^ = 21.13, *P* = 0.03, *I*^2^ = 48%; Figure 6A).

##### Apoptosis rate

Meta-analysis of 11 studies (Guo et al., 2006; Zhao et al., 2012; Cui G. et al., 2013; Yin et al., 2013; Hu et al., 2016; Meng et al., 2016; Wang et al., 2016, 2017; Gao et al., 2017; Zhang X. et al., 2017; Tang et al., 2018) showed DSS has significant effects on reducing apoptosis rate compared with controls (*n* = 74, SMD: −3.72, 95% CI: −4.93 to −2.51, *P* < 0.00001; heterogeneity χ^2^ = 17.38, *P* = 0.07, *I*^2^ = 42%; Figure 6B).

#### Cardioprotective Mechanisms

**Figure 7:**
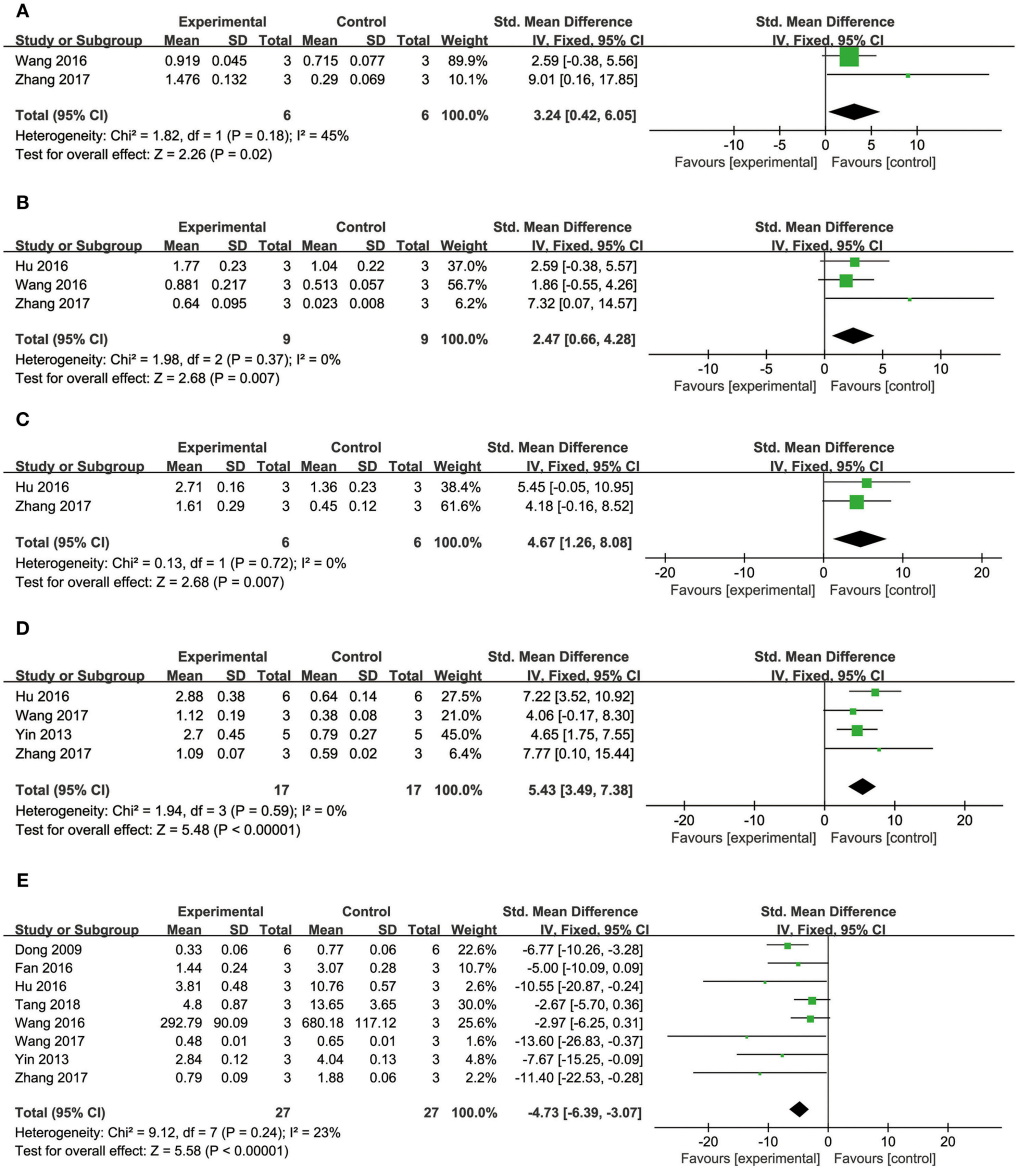
The forest plot: **(A)** *in vitro* effects of danshensu for increasing PGC1-α compared with controls (*n* = 6 per group); **(B)** *in vitro* effects of danshensu for increasing Nrf2 compared with controls (*n* = 9 per group); **(C)** *in vitro* effects of danshensu for increasing HO-1 compared with controls (*n* = 6 per group); **(D)** *in vitro* effects of danshensu for increasing Bcl-2/Bax compared with controls (*n* = 17 per group); **(E)** *in vitro* effects of danshensu for reducing caspase-3 compared with controls (*n* = 27 per group). PGC1-α, peroxisome proliferator activated receptor γ coactivator-1α; Nrf2, nuclear factor erythroid 2-related factor 2; HO-1, heme oxygenase-1; Bcl-2/Bax, B-cell lymphoma-2/BCL2-associated X protein.

**Figure 8:**
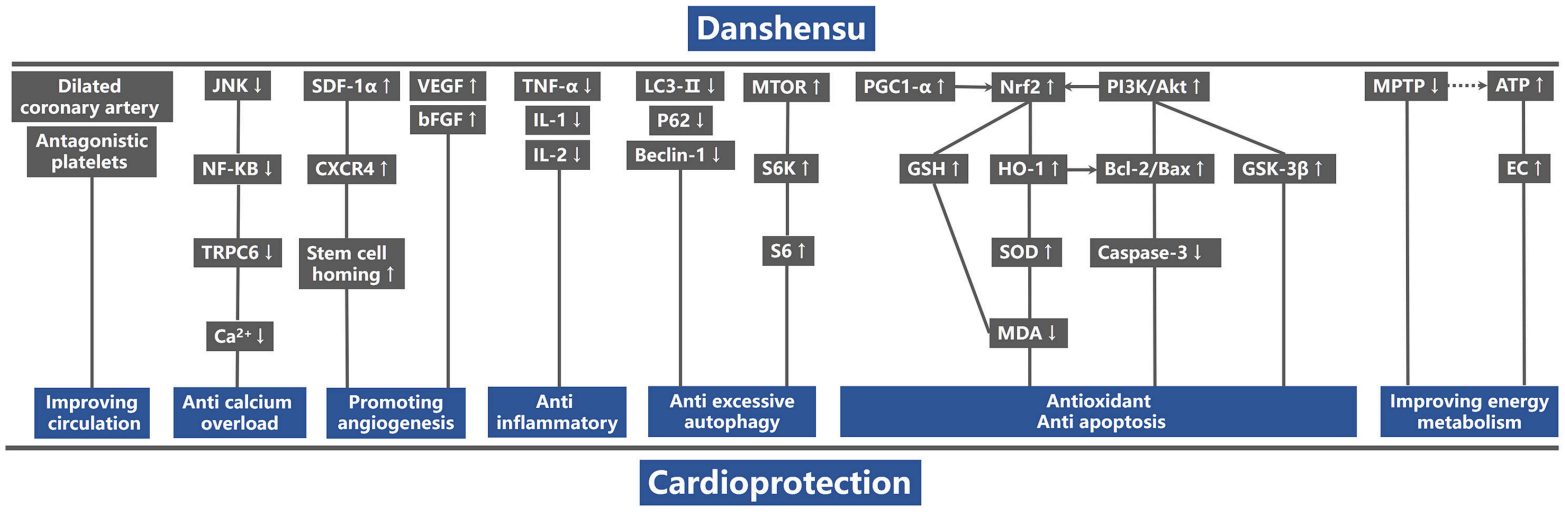
A schematic representation of cardioprotective mechanism of DSS for myocardial ischemic injury. P-Akt, phosphothreonine kinase; P-PI3K, phosphatidylinositol 3-kinase; P-GSK-3β, phosphorylated gGlycogen synthase kinase-3 beta; PGC1-α, peroxisome proliferator activated receptor γ coactivator-1α; Nrf2, nuclear factor erythroid 2-related factor 2; HO-1, heme oxygenase-1; GSH, glutathione synthetase; MDA, malondialdehyde; SOD, superoxide dismutase; Bcl-2, B-cell lymphoma-2; Bax, BCL2-associated X protein; TNF-α, tumor necrosis factor-α; IL-1, Interleukin-1; IL-6, Interleukin-6; SDF-1α, stromal cell-derived factor-1 α; CXCR4, C-X-C chemokine receptor type 4; VEGF, vascular endothelial growth factor; bFGF, basic fibroblast growth factor; P-mTOR, phosphorylated mammalian target of rapamycin; P-S6k1, phosphorylated ribosomal protein S6 kinase beta-1; P-S6, phosphorylated ribosomal protein s6; p62, sequestosome-1; LC3, microtubule-associated protein light chain 3; P-JNK, phosphorylated c-Jun N-terminal kinase; NF-KB, nuclear factor-κB; TRPC6, transient receptor potential cation channel, subfamily C, member 6; ATP, adenosine triphosphate; EC, energy charge; MPTP, Mitochondrial permeability transition pore. Solid lines indicate established effects, whereas dashed lines represent putative mechanisms.

Comparing DSS with controls, meta-analysis of 5 studies (Cui G. et al., 2013; Yin et al., 2013; Hu et al., 2016; Wang et al., 2016; Tang et al., 2018) showed significant effects on reducing LDH (*n* = 42, SMD: −4.08, 95% CI: −5.55 to −2.61, *P* < 0.00001; heterogeneity χ^2^ = 6.51, *P* = 0.16, *I*^2^ = 39%; Figure 6C); 1 study (Cui G. et al., 2013) for increasing p-PI3K (*P* < 0.05) and phosphorylated Glycogen synthase kinase-3 beta (p-GSK-3β) (*P* < 0.05); three studies (Cui G. et al., 2013; Yin et al., 2013; Hu et al., 2016) for increasing p-AKT (*n* = 28, SMD: 4.46, 95% CI: 2.68–6.24, *P* < 0.00001; heterogeneity χ^2^ = 2.89, *P* = 0.24, *I*^2^ = 31%; Figure 6D); two studies (Wang et al., 2016; Zhang X. et al., 2017) for increasing peroxisome proliferator activated receptor γ coactivator-1α (PGC1-α) (*n* = 12, SMD: 3.24, 95% CI: 0.42–6.05, *P* < 0.05; heterogeneity χ^2^ = 1.82, *P* = 0.18, *I*^2^ = 45%; Figure 7A); three studies (Hu et al., 2016; Wang et al., 2016; Zhang X. et al., 2017) for increasing Nrf2 (*n* = 18, SMD: 2.47, 95% CI: 0.66–4.28, *P* = 0.007; heterogeneity χ^2^ = 1.98, *P* = 0.37, *I*^2^ = 0%; Figure 7B); two studies (Hu et al., 2016; Zhang X. et al., 2017) for increasing HO-1 (*n* = 18, SMD: 4.67, 95% CI: 1.26–8.08, *P* < 0.00001; heterogeneity χ^2^ = 0.13, *P* = 0.72, *I*^2^ = 0%; Figure 7C); one study (Dong et al., 2009) for reducing MDA (*P* < 0.05); 1 study (Fan et al., 2016) for increasing Bcl-2 (*P* < 0.05) and reducing Bax (*P* < 0.05); four studies (Yin et al., 2013; Hu et al., 2016; Wang et al., 2017; Zhang X. et al., 2017) for increasing Bcl-2/Bax (*n* = 34, SMD: 5.43, 95% CI: 3.49–7.38, *P* < 0.00001; heterogeneity χ^2^ = 1.94, *P* = 0.59, *I*^2^ = 0%; Figure 7D); eight studies (Dong et al., 2009; Yin et al., 2013; Fan et al., 2016; Hu et al., 2016; Wang et al., 2016, 2017; Zhang X. et al., 2017; Tang et al., 2018) for reducing caspase-3 (*n* = 54, SMD: −4.73, 95% CI: −6.39 to −3.07, *P* < 0.00001; heterogeneity χ^2^ = 9.12, *P* = 0.24, *I*^2^ = 23%; Figure 7E); one study (Fan et al., 2016) for increasing p-mTOR (*P* < 0.01); 1 study (Meng et al., 2016) for reducing phosphorylated c-Jun N-terminal kinase (P-JNK) (*P* < 0.01), nuclear factor-κB (NF-KB) (*P* < 0.01) and transient receptor potential cation channel, subfamily C, member 6 (TRPC6) (*P* < 0.01). 2 studies (Zhu et al., 1999; Meng et al., 2016) for reducing Calcium (*P* < 0.01); We summarized a schematic representation of cardioprotective mechanism of DSS for myocardial ischemic injury (Figure 8).

## Discussion

### Summary of Evidence

This is the first preclinical systematic review to estimate the efficacy and mechanisms of DSS for Myocardial ischemic injury through both *in vivo* and *in vitro*. Thirty-two studies with 473 animals and 134 cells were selected. The quality of the included studies was generally moderate. The present study indicated that DSS exerted cardioprotective function in myocardial ischemic injury, mainly through improving circulation, antioxidant, anti-apoptosis, anti-inflammatory, promoting angiogenesis, anti-excessive autophagy, anti-calcium overload and improving energy metabolism.

### Limitations

First, only English and Chinese database were searched, which may lead to selective bias (Guyatt et al., 2011); Second, negative studies were defficult to publish, which may overstimate the true effecacy of DSS (Franco et al., 2014); Third, no study had used an animal with comobidity, such as diabetes, hypertension, or hyperlipidemia (Heusch, 2017); Finally, primary adult cardiomyocytes were considered to be the suitable subjects to validate cardioprotection *in vitro* (Lecour et al., 2014), but only one study (Zhu et al., 1999) used it.

### Implications

The damage inflicted on the myocardium during AMI is a complex process, which involves multiple factors. The main mechanisms of injury are energy metabolism disorders, excessive oxy radical, calcium overload, inflammation and mitochondrial dysfunction (Turer and Hill, 2010). Thus, cardiovascular protection drugs generally work through one or combined aspects of the above targets (Heusch, 2015; Ibanez et al., 2015; Qi and Young, 2015). The present study reveals that DSS can reduce MI size, increase cell viability, and reduce apoptosis in myocardial ischemic injury through multiple targets. Hence, our findings provide a preclinical evidence-based approach to develop DSS for acute MI. The possible mechanisms of DSS for myocardial ischemic injury are summarized as follows: (1) improving circulation by expanding the coronary artery and antiplatelet action; (2) antioxidant by promoting the Nrf2 accumulation in nucleus through activation of PI3K/AKTsignaling pathway; (3) anti-apoptosis via up-regulation of Bcl2/Bax and reducing the expression of caspase 3; (4) anti-inflammatory; (5) promoting angiogenesis by motivating the homing of stem cells and increasing the expression of VEGF; (6) anti-excessive autophagy via activation of m-TOR signaling pathway; (7) anti-calcium overload by reducing the expression of TRPC6 via inhibiting JNK/NF-KB signaling pathway; (8) improving energy metabolism.

Preclinical systematic review is a novel means that provides robust and informative evidence for animal researches. It contributed to decisions on the utility of further preclinical experiments. Additionally, important information about the translational probability of bench to bed was presented according to the preclinical evidence rating (Sena et al., 2014). In this study, the average CAMARADES score of the included studies is generally medium and still needs further improvement. For animal studies, the main defects are lacking of sample size estimation, blinded induction of model, and blinded assessment of outcome (Zhang K. J. et al., 2017). In addition, all the animal experiments are performed in young and healthy small animals which lack the comorbidities, such as aged, diabetes, hypertension and hyperlipidemia (Van Hout et al., 2016; Heusch, 2017). Cardioprotective effects from a longer-term perspective, such as chronic inflammation, remodeling, heart failure or mortality are not considered (Heusch, 2017). These are main failure factors to translate cardioprotective strategies from the animal study to the clinical practice (Lecour et al., 2014; Heusch, 2017). Reporting guidelines set a detailed checklist of predetermined criteria to make reports of biomedical research more complete and transparent, and thus increasing their value in Scientific exploration, clinical practice and inform policy (Moher et al., 2015). In 2010, the Animal Research: Reporting of *in vivo* Experiments (ARRIVE) guidelines were published on the initiative of the UK National Centre for the Replacement, Refinement and Reduction of Animals in Research (Kilkenny et al., 2010). The ARRIVE guidelines are organized into twenty sections, covering the key points on reporting study design, experimental procedures, and experimental animals to improve comprehensive and transparent on reporting (Karp et al., 2015). Items 6 and 10 of ARRIVE guideline emphasizes the importance of sample size calculation and blinding. Therefore, we suggest the further animal studies should refer to the ARRIVE and sample size estimation, blinded induction of model, and blinded assessment of outcome should be focused on. In addition, the following factors need to be considered: (1) using large mammals that are closer to humans in anatomy and physiology; (2) experimental animals have comorbidities with aged, diabetes, hypertension, hyperlipidemia or other risk factors; (3) use of primary outcome measures that are closer to clinical practice.

For cells, there are lacking of randomization and blinding (Zhang K. J. et al., 2017). In addition, primary adult cardiomyocytes were considered to be the suitable subjects to validate cardioprotection *in vitro* (Lecour et al., 2014), however, only one study (Zhu et al., 1999) used it. Thirdly, most cell experiments mimicked the acute I/R injury model using compounds, such as t-BHP, which cannot reflect the complex conditions *in vivo* (Lecour et al., 2014). Thus, we suggest the further studies focused on randomization and blinding in cell experiments, and it is best to use primary adult cardiomyocytes in ischemic buffer with oxygen-deficient environment, then mimics perfusion using normal buffer with normoxic environment.

Cochrane ROB tool that designed for evaluating the quality of clinical trials was used worldwide (O'Connor et al., 2011). Similarly, some tools have been developed to evaluate the quality of preclinical animal experiments *in vivo* (Hooijmans et al., 2014; Zhang K. J. et al., 2017). However, there are no quality assessment tools for cell experiments. Thus, we developed an evaluation tool for quality of cell experiments. Detailed description of the assessment tool is as follows: (1) peer review is an inseparable part of science. Non-peer reviewed articles are considered to be of inferior quality and negligible value (Costello et al., 2013). Therefore, it is necessary to use peer review as a part of quality assessment (Table 5A: peer reviewed publication); (2) The use of cell models to simulate clinical disease is important in modern research. However, they do not accurately represent organisms and cannot reproduce the complex interactions *in vivo*. Thus, it is necessary to select cells that can maximally simulate clinical disease. For example, primary adult cardiomyocytes are most closely related to the characteristics of the adult heart in cell experiments (Lecour et al., 2014). Hence, in the present study, they are the appropriate cells for simulating myocardial ischemic injury. Here we call on researchers to choose the appropriate cells based on actual situation (Table 5B: use appropriate cells to study); (3) quality control of cell lines is essential to ensure the reproducibility of biomedical research. Although cell line certification has been widely recommended for many years, misidentification and cross-contamination are still a serious problem. *Science* and *nature*, the two top journals, also have repeatedly called attention to this issue (Freedman et al., 2015; Neimark, 2015; Yu M. et al., 2015). Thus, we recommend using cell lines with reliable source or validated by appropriate methods (Table 5C: cell lines with reliable source or validated by appropriate methods); (4) efforts to improve the safety of drugs have always been the key to pharmacological research (Kalgutkar and Dalvie, 2015; Nussinov and Tsai, 2015; Insel et al., 2018). Therefore, special attention should be paid to the toxicity of treatment in preclinical studies (Table 5D: assess toxicity of treatment on cells). (5) The culture environment is crucial for cell growth and subsequent experiments. It is necessary to select a suitable culture environment according to the cell species [Table 5E: culture environment (culture media/sera, pH/CO_2_ and temperature)]; (6) the items of random allocation and blinding are essential to RoB (O'Connor et al., 2011; Zhang K. J. et al., 2017) (Table 5F: random allocation to treatment or control; 5G: blinded induction of model; 5H: blinded assessment of outcome); (7) experiments should have enough sample size to allow for proper statistical analysis and to ensure that the results are robust and reliable. But to avoid waste of resources and prevent exposure of research participants to risk associated with the interventions, the calculation of the sample size necessary to achieve sufficient power is needed (Stokes, 2014) (Table 5I: calculation of the sample size necessary to achieve sufficient power); (8) conflicts of interest are one source of bias. They exist when professional judgment or actions regarding a primary interest may be unduly influenced by a secondary interest (Bero, 2017) (Table 5J: statement of potential conflict of interests).

Improving the transparency and quality of reporting is the imperative of modern scientific research. Numerous publications have emphasized the lack of transparency in reporting, but researches in life sciences, still often lack reporting on the design, conduct and analysis of the experiments (Landis et al., 2012). This resulted in the Consolidated Standards of Reporting Trials (CONSORT) Statement, which was first published in 1996 (Begg et al., 1996), and had been proven to improve the transparency of clinical researches in subsequent implementation (Plint et al., 2006). In 2004, CAMARADES 10-item checklist was first developed to assess the quality of animal experiments (Macleod et al., 2004). Subsequently in 2012, CAMARADES called for transparent reporting to optimize the predictive value of preclinical research (Landis et al., 2012). They recommend that, at minimum, a rigorous study design consist of randomization, blinding, sample-size estimation and the handling of all data should be reported. In the present study, we emphasized the importance of the above recommendations, the items F, G, H, and I in the cell study checklist are consistent with the CAMARADES claim. In addition, misidentification, cross-contamination, cytotoxicity, and culture environment are core issues widely existed in cell studies. Therefore, we recommend that authors should report on exact source of the cells, the method of verifying the cell type, toxic effect of the drug on the cells, and the detailed data of the cell culture environment. We hope and expect that this tool will fill in the blank areas for preclinical systematic review and meta-analysis of cell studies, subsequently improving the transparency and quality of the *in vitro* experiments. Furthermore, this tool should be tested for its applicability and validity in practice. We look forward to the feedback from users on this new field of evidence-based cell experiments to make this tool more rigorous, scientific, and valuable.

## Conclusion

The findings of present study demonstrated that DSS exerted cardioprotective function in AMI, largely through improving circulation, antioxidant, anti-apoptosis, anti-inflammatory, promoting angiogenesis, anti-excessive autophagy, anti-calcium overload and improving energy metabolism. Thus, DSS is a probable candidate for further AMI treatment and clinical trials. In addition, the newly devloped 10-item checklist for assessing methodological quality of cell study that recommened to use the sysmatic review and meta-analysis of cell studies.

## Author Contributions

XB and QZ contributed equally to this work. XB, QZ, QT, PZ, ZZ, GZ, and YW designed the study. QT, PZ, and ZZ collected the data. XB, QZ, and QT performed all analyses. XB and QZ wrote the manuscript.

### Conflict of Interest Statement

The authors declare that the research was conducted in the absence of any commercial or financial relationships that could be construed as a potential conflict of interest.
